# Phosphorylated and sumoylation-deficient progesterone receptors drive proliferative gene signatures during breast cancer progression

**DOI:** 10.1186/bcr3211

**Published:** 2012-06-14

**Authors:** Todd P Knutson, Andrea R Daniel, Danhua Fan, Kevin AT Silverstein, Kyle R Covington, Suzanne AW Fuqua, Carol A Lange

**Affiliations:** 1 Departments of Medicine (Division of Hematology, Oncology, and Transplantation) and Pharmacology, Masonic Cancer Center, University of Minnesota, 420 Delaware Street SE, Minneapolis, MN 55455 USA; 2 Biostatistics and Bioinformatics Core, Masonic Cancer Center, 425 Delaware St SE, University of Minnesota, Minneapolis, MN 55455 USA; 3 Department of Medicine, Lester and Sue Smith Breast Center, Baylor College of Medicine, One Baylor Plaza, Houston, TX 77030 USA

## Abstract

**Introduction:**

Progesterone receptors (PR) are emerging as important breast cancer drivers. Phosphorylation events common to breast cancer cells impact PR transcriptional activity, in part by direct phosphorylation. PR-B but not PR-A isoforms are phosphorylated on Ser294 by mitogen activated protein kinase (MAPK) and cyclin dependent kinase 2 (CDK2). Phospho-Ser294 PRs are resistant to ligand-dependent Lys388 SUMOylation (that is, a repressive modification). Antagonism of PR small ubiquitin-like modifier (SUMO)ylation by mitogenic protein kinases suggests a mechanism for derepression (that is, transcriptional activation) of target genes. As a broad range of PR protein expression is observed clinically, a PR gene signature would provide a valuable marker of PR contribution to early breast cancer progression.

**Methods:**

Global gene expression patterns were measured in T47D and MCF-7 breast cancer cells expressing either wild-type (SUMOylation-capable) or K388R (SUMOylation-deficient) PRs and subjected to pathway analysis. Gene sets were validated by RT-qPCR. Recruitment of coregulators and histone methylation levels were determined by chromatin immunoprecipitation. Changes in cell proliferation and survival were determined by 3-(4,5-dimethylthiazol-2-yl)-2,5-diphenyltetrazolium bromide (MTT) assays and western blotting. Finally, human breast tumor cohort datasets were probed to identify PR-associated gene signatures; metagene analysis was employed to define survival rates in patients whose tumors express a PR gene signature.

**Results:**

'SUMO-sensitive' PR target genes primarily include genes required for proliferative and pro-survival signaling. DeSUMOylated K388R receptors are preferentially recruited to enhancer regions of derepressed genes (that is, *MSX2*, *RGS2*, *MAP1A*, and *PDK4*) with the steroid receptor coactivator, CREB-(cAMP-response element-binding protein)-binding protein (CBP), and mixed lineage leukemia 2 (MLL2), a histone methyltransferase mediator of nucleosome remodeling. PR SUMOylation blocks these events, suggesting that SUMO modification of PR prevents interactions with mediators of early chromatin remodeling at 'closed' enhancer regions. SUMO-deficient (phospho-Ser294) PR gene signatures are significantly associated with human epidermal growth factor 2 (ERBB2)-positive luminal breast tumors and predictive of early metastasis and shortened survival. Treatment with antiprogestin or MEK inhibitor abrogated expression of SUMO-sensitive PR target-genes and inhibited proliferation in BT-474 (estrogen receptor (ER)+/PR+/ERBB2+) breast cancer cells.

**Conclusions:**

We conclude that reversible PR SUMOylation/deSUMOylation profoundly alters target gene selection in breast cancer cells. Phosphorylation-induced PR deSUMOylation favors a permissive chromatin environment via recruitment of CBP and MLL2. Patients whose ER+/PR+ tumors are driven by hyperactive (that is, derepressed) phospho-PRs may benefit from endocrine (antiestrogen) therapies that contain an antiprogestin.

## Introduction

Breast cancer is the most commonly diagnosed cancer in women, and the second leading cause of cancer-related death [1]. The molecular factors driving its initiation and progression are not completely understood. A randomized clinical trial by the Women's Health Initiative (WHI) demonstrated that hormone replacement therapy (HRT), containing estrogens and progestins (but not estrogens alone), significantly increased the risk of developing invasive breast cancer in post-menopausal women [2,3]. A similar conclusion was made from the Million Women observational study [4]. These findings resulted in dramatically fewer prescriptions for HRT and, as a result, breast cancer incidence dropped considerably [5]. Further analysis of the WHI data demonstrated that women prescribed HRT containing estrogens alone experienced a reduced risk of developing invasive breast cancer [3,6]. Progesterone receptor (PR) expression is traditionally used as a clinical indicator of estrogen receptor (ER) function (that is, PR is an ER target gene). However, while controversial, this surprising epidemiological evidence provides a strong rationale for further investigation of the unique actions of PRs as mediators of breast cancer initiation and early progression (reviewed in [7]).

Classically, PRs are defined as ligand-activated transcription factors that bind target gene promoters or enhancers as dimers capable of recruiting coregulatory molecules required for efficient transcription. More recently, it has become well recognized that protein kinases are rapidly activated by steroid hormones (as in response to peptide growth factors). Indeed, phosphorylation events provide key regulatory inputs to PR action (reviewed in [8] and discussed below). A few mutations in PR have been linked to cancer risk; these appear to primarily alter PR expression levels rather than impact PR transcriptional activity [9-11]. Two PR protein isoforms, PR-A and PR-B, are co-expressed in breast tissues. PR-B is the full-length receptor, containing 164 amino acids at the N-terminus (termed the B-upstream segment or BUS) that are absent from PR-A. Both isoforms are heavily post-translationally modified (phosphorylation, ubiquitination, acetylation). PR N-termini contain key regulatory phosphorylation sites (for example, Ser294) as well as a SUMOylation site (Lys388) investigated herein. PR-B, but not PR-A, is phosphorylated on Ser294 in cell culture and *in vivo *[12]. Upon ligand binding, both PR isoforms are rapidly (15 minutes) SUMOylated at Lys388 [13]. SUMOylation occurs via the covalent attachment of a small ubiquitin-like modifier (SUMO) peptide (approximately 11.5 kD) to lysine residues of substrate molecules, primarily at consensus SUMOylation motifs (IKxE) through an ATP-dependent enzymatic (three step) mechanism, similar to that of ubiquitination [14]. Substrate SUMOylation often alters protein-protein interactions, subcellular location, protein stability (that is, it can oppose ubiquitination), and/or enzyme or transcriptional activities [15].

Recently, Daniel *et al. *discovered that PR-B phosphorylation at Ser294, in response to activated mitogen activated protein kinases (MAPKs) or cell cycle-dependent protein kinase-two (CDK2), prevents progestin-induced rapid SUMOylation at Lys388 [13,16]. Additionally, Ser294 phosphorylation-induced antagonism of PR SUMOylation derepressed (activated) PR transcriptional activity at selected breast cancer-associated gene promoters, namely *HBEGF *[13], *STC1 *and *IRS1 *[16]; phospho-PR-dependent upregulation of the breast cancer-associated drivers, *STC1 *and *IRS*, occurred in the absence of progestins [16]. Promoter structure (that is, the number of hormone response elements) is a key determinant of reporter-gene promoter recognition by SUMOylated glucocorticoid receptors (GRs) [17], while much less is known about how steroid receptor (SR) SUMOylation alters the regulation of endogenous genes (that is, in chromatin). To date, only a few endogenous genes have been shown to be sensitive to PR SUMOylation [13,16]. We propose that PR acts as a sensor for activated mitogenic protein kinases (that is, MAPKs and CDK2) frequently elevated in human breast cancer; under the influence of elevated Ser294 phosphorylation, genes that are sensitive to (that is, normally repressed by) PR SUMOylation may instead cooperate to drive breast cancer cell proliferation and pro-survival signaling. A phospho-PR (SUMO-deficient) gene signature may identify a subset of human breast cancer patients likely to respond to endocrine therapies that contain a selective antiprogestin.

We addressed mechanisms of PR promoter selectivity related to dynamic post-translational events (that is, PR Ser294 phosphorylation coupled to Lys388 deSUMOylation). We employed whole genome expression analysis to identify genes that are differentially regulated by wild-type (WT) and SUMO-deficient (K388R) PR-B and explored the mechanisms responsible for altered PR promoter selectivity. Our findings implicate SUMO-deficient phospho-PR-B in the selective regulation of genes that are important for breast cancer cell proliferation and are pro-survival, and suggest that phosphorylated and deSUMOylated PRs may be important drivers of the ERBB2+ phenotype associated with rapid (luminal) breast cancer tumor progression.

## Materials and methods

### Progesterone receptor expression in human breast tumor samples

De-identified human breast tumor samples were obtained from the University of Minnesota Tissue Procurement Facility's Biological Materials Procurement Network (BioNet) for protein and mRNA analysis. Frozen tissue samples were derived from patients diagnosed with either ductal carcinoma, infiltrating ductal carcinoma, lobular carcinoma, or metastatic carcinoma. Specimens were analyzed by the University of Minnesota clinical pathology department and scored for ER and PR expression using standard clinical histological methods. Tumor samples were harvested individually for protein or mRNA using standard methods (frozen tissue grinding, radioimmunoprecipitation assay (RIPA) buffer, tri-reagent), and total PR, phospho-Ser294 PR and ERK1/2 protein expression levels were measured by western blotting (described below). All specimens were obtained from patients with informed consent and approval from University of Minnesota Institutional Review Board (IRB).

### Cell culture, expression vectors and western blotting

T47Dco parental cell lines were characterized previously [18]. T47D cells stably expressing PR were created by molecular cloning of cDNAs encoding either WT, K388R, S294A, or K388R/S294A PR into a pIRES-neo3 expression vector (Clontech, Mountain View, CA, USA, catalog #631621), followed by transfection of vectors into T47D-Y cells [19] using FuGENE HD (Roche, Indianapolis, IN, USA, catalog #04709713001). Single-cell clones were expanded under high G418 selection (500 ug/ml) and maintained in low G418 selection (200 ug/ml) (EMD Chemicals, Billerica, MA, USA, catalog #345810). These cells were maintained in complete minimal essential medium (cMEM) supplemented with 5% fetal bovine serum (FBS), 1% non-essential amino acids (NEAA), 1% penicillin/streptomycin, 6 ng/ml insulin (CellGro, Manassas, VA, USA, catalog #10-010-CV). T47D cells expressing inducible PR were described previously [20]. Inducible PR expression was achieved by adding AP21967 (10^-9 ^M, Ariad Pharmaceuticals, Cambridge, MA, USA) to cell culture medium for a minimum treatment time of two days. MCF-7 cell lines expressing PR were created by transfection of pIRES-neo3 vectors containing cDNA inserts encoding either WT or KR PR into cells using FuGENE HD. Single-cell clones were expanded under high G418 selection and maintained in low G418 selection. MCF-7 cells were maintained in (D)MEM (CellGro, catalog #10-013-CV) supplemented with 5% FBS, 1% penicillin/streptomycin. BT-474 cells (ATCC, Manassas, VA, USA) were maintained in Roswell Park Memorial Institute (RPMI) 1640 medium (Gibco, Grand Island, NY, USA, catalog #11875) supplemented with 10% FBS, 1% penicillin/streptomycin. SDS-PAGE was performed using 8% gels and western blotting analysis was performed as previously described [13]. For antibody information, see Additional file 1.

### Gene expression profiling

**Figure 1:**
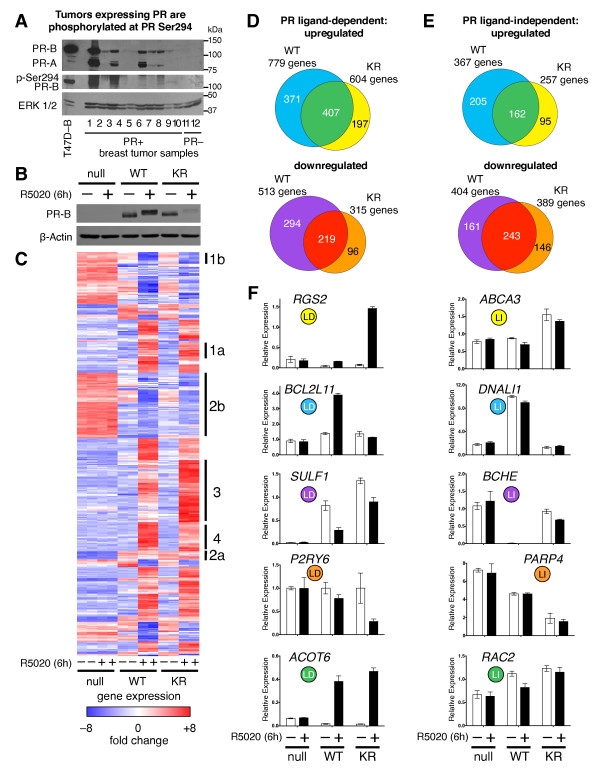
**Gene expression profiling of T47D cells stably expressing WT or SUMO-deficient PR, treated with or without R5020 for six hours**. (**A**) Western blot showing total and phospho-Ser294 PR proteins (total ERK1/2 served as a loading control) in 12 human breast tumors. (**B**) T47D cells stably expressing either wild-type PR-B (WT), SUMO-deficient mutant K388R PR-B (KR), or empty vector (null) controls were treated without or with R5020 prior to western blotting for PR-B. (**C**) Heat map showing normalized expression values for differentially expressed transcripts (fold change > 8.0 in at least one sample, BH adjusted *P *< 0.001). Biological duplicates are shown for each treatment group and notable gene expression categories (numbered 1-4 on right side) are described (see Results). (**D**) Venn diagrams showing up- or down-regulated PR target genes following progestin treatment (log_2 _fold change > 0.6, BH adjusted *P *< 0.01; common fold change > 1.5). (**E**) Venn diagrams (as in part D) depicting the number of ligand-independent (LI) PR target genes up- or down-regulated relative to PR-null cells. (**F**) Relative mRNA expression (as determined by RT-qPCR) of selected PR target genes in T47D cells stably expressing vector control (PR-null), WT or KR PR and treated without or with R5020 for six hours; genes chosen from ligand-dependent (LD) or LI Venn categories are indicated (note matching color labels). Data are represented as mean of *n *= 3 +/- SD. [See also Additional file 5. BH, Benjamini and Hochberg; n, number; PR, progesterone receptor; SD, standard deviation; SUMO, small ubiquitin-like modifier.

T47D cells stably expressing pIRES-neo3 empty vector, WT or KR PR were serum starved in modified improved MEM (IMEM (Gibco, catalog #A10488) for one day, treated with R5020 (10^-8 ^M) or vehicle control for six hours before RNA extraction using a RNeasy kit (QIAgen, Germantown, MD, USA, catalog #74104). Six hours of progestin treatment allowed for substantial PR-dependent gene expression as compared to prior studies [21,22]. DNase I treated (QIAgen, catalog #79254) RNA samples from duplicate experiments were prepared for expression analysis using the Illumina HT-12v4 bead chip platform (San Diego, CA, USA) according to the manufacture's protocols. Data were analyzed within R software [23] using the Bioconductor [24] package called lumi where raw intensities were log_2 _transformed and quantile normalized. Differentially expressed genes were analyzed using the limma package, where empirical Bayes was used to better estimate the variance of the genes. Gene expression data presented contain log_2 _normalized intensities and biological comparisons presented (for example, R5020/vehicle) contain log_2 _fold change with the Benjamini and Hochberg (BH) adjusted *P *value [25]. To generate the heat map in Figure 1C, unsupervised hierarchical clustering of genes was carried out using the heatmap.2 function in the R package gplots. Clustering was performed using Euclidean distance and complete linkage. Rows were scaled to have mean zero and standard deviation equal to one.

Gene expression profiles in T47D cells expressing inducible PR were measured using the Affymetrix microarray platform (Santa Clara, CA, USA). PR expression was induced with AP21967 (10^-9 ^M) for two days, cells were serum starved in modified IMEM for one day and treated with R5020 (10^-8 ^M) or vehicle control for six hours before RNA extraction using an RNeasy kit. DNase I treated samples were prepared for expression analysis using the Affymetrix U133A 2.0 microarrays according to the manufacture's protocols. Raw Affymetrix CEL files were processed and normalized within R using the Bioconductor [24] packages, affy and affyQCReport. Data were normalized using the Robust Multi-array Average [26] algorithm within the affy package. Wilcoxon-signed rank tests as part of the MAS 5.0 algorithm (also included in the affy package) were used to determine presence/absence calls for all probe sets [27]. Normalized expression levels for selected pairs of conditions were computed as log_2 _ratios. All gene expression data is available in the NCBI Gene Expression Omnibus (GEO) database (accession number: GSE34149).

### RT-qPCR

For reverse transcription quantitative polymerase chain reaction (RT-qPCR) assays, 5 × 10^5 ^cells/well were plated in six-well dishes, serum starved in modified IMEM for one day before treatments (see individual figures). RNA was extracted using TriPure reagent (Roche, catalog #11667157001) and cDNA was created using the Transcriptor cDNA first-strand cDNA synthesis kit (Roche, catalog #04897030001). Relative expression levels were determined by qPCR assays performed on a Roche LightCycler II using SYBR green master-mix (Roche, catalog #04887352001). Target gene quantification levels were normalized to the expression of standard housekeeper genes: *TBP*, *ACTB*, and/or *GAPDH*. For cells expressing inducible PR, the protocol was the same as above, except prior to ligand treatments, the cells were induced with AP21967 (10^-9 ^M) for two days.

For RT-qPCR assays involving epidermal growth factor (EGF) treatment, cells were plated at 5 × 10^5 ^cells/well in six-well dishes and serum starved for two days in modified IMEM. Cells were pre-treated with 100 ng/ml EGF (Sigma, St. Louis, MO, USA, catalog #E9644) before treatment with R5020 (10^-8 ^M).

For experiments using MEK inhibitors, BT-474 cells were plated in six-well dishes at 5 × 10^5 ^cells/well. One day later, the cells were washed and serum starved in modified IMEM for one day. These cells were pre-treated with the MEK inhibitor U0126 (5 uM, EMD Chemicals, catalog #662005) for 30 minutes. R5020 (10^-8 ^M) and/or RU486 (10^-7 ^M) was then added to the cell culture wells for six hours before RNA/protein isolation and RT-qPCR/western blotting was performed, as described above. PCR primer sets used in this study are provided in Additional file 1.

### Ingenuity Pathway Analysis

Ingenuity Pathway Analysis (IPA) was used to compare two distinct gene lists: those upregulated by progestin in T47D cells expressing WT PR compared to genes upregulated by progestin in cells expressing SUMO-deficient PR [See Additional file 2, +R5020/-R5020 log_2 _fold change > 1.0, BH adjusted *P *< 0.01]. These gene lists were uploaded into the IPA software where a core analysis was completed to determine the association of each gene with various biological functions or network pathways. IPA comparison analyses were used to reveal whether or not cells expressing WT or KR PR upregulated functionally distinct pathways. Analyses were scored based on significance (the BH adjusted *P *value, corrected for multiple hypothesis testing) and the threshold for a gene list to be significantly involved in a particular biological function was *P *< 0.05 (or -log_10_(BH adjusted *P *value) > 1.30).

### Identification of PR expression metagenes

Metagene analysis was conducted using gene expression microarray data from cell lines constitutively expressing empty vector, WT PR, or K388R PR, and treated with either vehicle or R5020. A strategy of identifying metagenes within each sample was employed using non-negative matrix factorization [28]. This strategy facilitated identification of metagenes and application to other datasets. To limit the study to genes under high variance and to limit the number of probes used in calculating the metagene fit, probes were considered for metagene analysis based on the interquartile range (IQR) of the probe being in the upper 80th percentile. The optimum rank of the data was calculated as eight; therefore, eight metagenes are present in the data. Three of these metagenes were either highly expressed in all samples, or expressed in no samples, indicating that they are likely metagenes for housekeeping or continually expressed genes. The remaining five metagenes corresponded to the empty vector PR-null samples (with no distinction between the -R5020 and +R5020 treatment), and the pairwise combination of WT or KR PR, with or without R5020. Thus, these analyses identified metagenes from biologically relevant subtypes of cells.

The Loi *et al. *human breast tumor dataset [29] contains gene expression data for both tamoxifen treated and untreated samples across several datasets. These data were aggregated together and are available through the gene expression omnibus (GEO) (accession number GSE6532). The dataset [29] was loaded into Red-R [30] for processing. The basis matrix for the metagene analysis was reshaped to aggregate across the gene symbols and average the metagene values across each probe of the gene (average value). The same manipulation was performed on the expression data. Non-matching genes (those that were present in the metagene data but not in the clinical expression data or vice versa) were removed from analysis. The reshaped data were supplied to the nonnegative matrix factorization (NMF) package function (fcnnls) for scoring (as was done to generate the initial metagene fit on the T47D cell line data). As the Loi *et al. *data are supplied as z-scores, the data were un-logged and used in the fcnnls algorithm (as they contain negative numbers in their normal form). Samples were taken to express a metagene if they showed a non-zero value in the fitted coefficient matrix (scoring matrix).

### Identification of novel PR-target genes and comparison analysis of gene expression platforms

Ligand-dependent and -independent PR-target gene lists from two previously published studies [21,22] were combined (duplicates were removed). Genes identified herein were upregulated (> 1.5 fold BH adjusted *P *< 0.01) as measured using either platform (Illumina and Affymetrix were combined) and duplicates were removed before Venn diagram comparison to previously known upregulated genes using the bioinformatics tool, VENNY [31].

Gene set enrichment analysis (GSEA) software [32,33] was employed to compare genes up- or down-regulated in cells stably expressing WT or KR PR to cells expressing inducible iWT or iKR PR [See Additional file 3]. Using the Affymetrix expression data, four gene sets were created: genes up- or down-regulated > 2.0 fold by iWT with R5020, and genes up- or down-regulated > 2.0 fold by iKR with R5020. Similarly, two GSEA-formatted datasets were created from the Illumina expression data: the first dataset compares the two phenotypes (WT +R5020 versus WT -R5020), and the second compares the two phenotypes (KR +R5020 versus KR -R5020). GSEA was performed using those Illumina datasets and queried for enrichment of the Affymetrix gene sets. GSEA was executed using the default settings, except the permutation type was set to Gene_set with 1,000 permutations, and the metric for ranking genes was set to Diff_of_Classes because our dataset contained log-scale data.

### Chromatin immnunoprecipitation

Chromatin immunoprecipitation (ChIP) assays were performed according to the ChIP-IT Express instruction manual (Active Motif, Carlsbad, CA, USA, catalog # 53008). Cells were plated at 15 × 10^6 ^cells per 15 cm culture dish in cMEM for two days, then serum starved in modified IMEM for two days. Cells were treated with R5020 (10^-8 ^M) or vehicle for one or four hours. For T47D cells expressing inducible PR, AP21967 (10^-9 ^M) was added during the starvation step. Chromatin was sheared using a Bioruptor sonicator (Diagenode, Denville, NJ, USA, model UCB-200), for 30 minutes (30 seconds on/off). Immunoprecipitations were prepared with 60 ul of sheared chromatin, 2 ug antibody and immunoprecipitated overnight. Using the purified ChIP and input DNA, relative recruitment was determined by qPCR in triplicate. Assays were performed on a Roche LightCycler II using SYBR green master-mix. Target locus quantification was normalized as a percentage of the input DNA quantification.

To assay H3K4me2 levels, nucleosomes were isolated using micrococcal nuclease (MNase). In 15 cm dishes, 12 × 10^6 ^cells were plated in cMEM, serum starved in modified IMEM and induced with AP21967 (10^-9 ^M) treatment for two days. One day later, cells were treated with R5020 (10^-8 ^M) for four hours and chromatin was harvested and immunoprecipitated as previously described [34].

### Cell proliferation and apoptosis assays

Cell proliferation was measured using MTT assays (3-[4,5-dimethylthiazol-2-yl]- 2,5-diphenyltetrazolium bromide, Sigma catalog #M2128). In 24-well plates, 1 × 10^4 ^cells/well were plated in cMEM (inducible PR expression was induced with AP21967 (10^-9 ^M) for two days), cells were washed and steroid starved in modified IMEM supplemented with 5% dextran-coated charcoal-treated (DCC) FBS for one day before the addition of R5020 (10^-8 ^M). At days 0, 2, 4, and 6, cell proliferation was determined by adding 60 ul MTT (5 mg/ml) to each 0.5 ml cell culture well for three hours, medium was carefully removed and solubilization solution (90% v/v dimethyl sulfoxide (DMSO)/PBS) was added to lyse the cells. Lysate absorbance (650 and 570 nm) was measured using a plate reader. The 650 nm measurements were subtracted from 570 nm measurements and sample means were normalized to day zero.

Poly (ADP)-ribose polymerase 1 (PARP) cleavage assays were used to measure the level of apoptosis in cell cultures after treatment with cytotoxic concentrations of doxorubicin. T47D cells expressing inducible PR were plated in 10 cm dishes (2 × 10^6 ^cells/dish) in cMEM and induced with AP21967 (10^-9 ^M). Cells were washed, induced, and serum starved for four days. Cells were then treated with R5020 (10^-8 ^M) for six hours before adding doxorubicin (8 uM) to dishes for 24 hours. Protein was harvested using standard RIPA lysis buffer, subjected to SDS-PAGE and western blotting using cleaved-PARP and PR antibodies. Beta-actin western blotting was performed for sample loading controls.

Cell viability after treatment with cytotoxic doxorubicin was determined by measuring the concentration of ATP, which is directly proportional to viable cell number [35], using Cell-Titer-Glo bioluminescence assays (Promega, Madison, WI, USA, catalog #G7571). T47D cells expressing WT or KR PR were plated in 24-well dishes (1 × 10^4 ^cells/well) containing cMEM. Cells were washed and steroid starved in modified IMEM supplemented with 5% DCC FBS for one day. Cells were treated with R5020 (10^-8 ^M) for six hours before doxorubicin (6 uM) was added to the wells. After four days, cell viability was determined by adding Cell-Titer-Glo substrate and luminescence was measured using a plate reader. Sample means were normalized to day zero (*n *= 6, -/+ standard deviation (SD)).

### Oncomine data analysis

**Figure 2:**
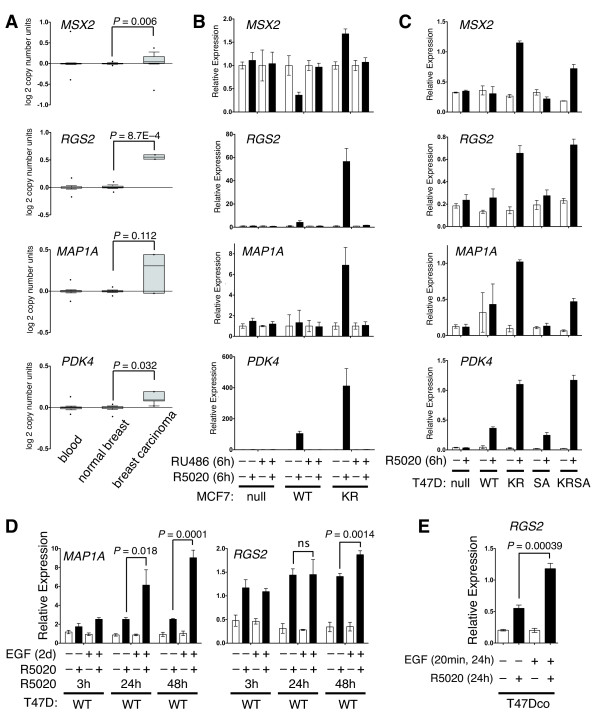
**Phosphorylation of PR Ser294 drives SUMO-deficient PR gene expression and promoter selectivity in MCF-7 and T47D cells**. (**A**) Relative expression level (copy number) of PR target genes in breast cancer patient cohorts. (**B**) Relative gene expression levels of selected PR target genes in MCF-7 cells stably expressing either empty vector (PR-null), WT or SUMO-deficient K388R PRs. Cells were co-treated with progestin R5020 and/or antiprogestin RU486 for six hours and mRNA levels were measured using RT-qPCR (see Materials and methods). (**C**) Relative gene expression levels of the same PR target genes (as in parts A-B) were measured using RT-qPCR in five vector-matched T47D cell lines stably expressing PRs: empty vector (null), wild-type (WT) PR, K388R mutant (KR) PR, S294A mutant (SA) PR, and K388R and S294A double mutant (KRSA) PR. Cells were treated with R5020 for six hours. (**D**) T47D cells expressing WT PR were treated cells with epidermal growth factor (EGF) for two days and treated with R5020 for 3, 24, or 48 hours. Relative *MAP1A *and *RGS2 *mRNA levels were measured using RT-qPCR. (**E**) Parental T47Dco cells were pretreated with EGF for 20 minutes prior to 24 hours of R5020 treatment. Relative *RGS2 *mRNA levels were measured by RT-qPCR. Data are represented as mean of *n *= 3 +/- SD and significance calculated using Student's t-test. n, number; PR, progesterone receptor; SD, standard deviation; SUMO, small ubiquitin-like modifier; WT, wild type.

The relative expression of individual PR target genes in human breast tumor samples was determined by searching the Oncomine database (version 4.4, October 2011 data release, [36]). Individual PR target genes (for example, *RGS2*) were queried in The Cancer Genome Atlas (TCGA) Breast 2 dataset. Oncomine output data was sorted to isolate 'cancer versus normal' associations and reported (Figure 2A) as the copy number unit expression values for blood, normal breast and breast carcinoma samples using box-and-whiskers plots (dots: maximum/minimum, whiskers: 90/10 percentiles, box: 75/25 percentiles, line: median of all samples). For each analysis, specific breast carcinomas specified for each gene are: Invasive Lobular Breast Carcinoma (*MSX2*), Invasive Ductal and Lobular Carcinoma (*RGS2*), Intraductal Cribriform Breast Adenocarcinoma (*MAP1A*), and Mucinous Breast Carcinoma (*PDK4*).

Multiple breast cancer 'concepts', as described in the Oncomine database, were associated with the ligand dependent (LD) KR > WT gene signature [See Additional file 4]. According to Oncomine, concepts are derived from gene expression microarrays or gene-copy-number datasets derived from tumor cohorts or cancer cell line experiments. Specifically, concepts are a list of genes from various published datasets that are defined by some criteria (for example, top 5% of genes expressed in ERBB2-positive breast tumors). The LD gene signature was created by normalizing the gene expression values in the +R5020 treatment group to the -R5020 treatment group, then comparing those normalized fold change values between the KR and WT PR expressing cell lines. This analysis identified 151 LD genes upregulated > 1.5 fold in cells expressing SUMO-deficient PR versus WT PR expressing cells. The ligand-independent (LI) gene signature was created by normalizing the gene expression values in -R5020 treatment group in WT or KR expressing cells to the -R5020 treatment group in the PR-null expressing cells, then comparing those normalized fold change values between the KR and WT expressing cell lines. This analysis identified 92 LI genes upregulated > 1.5 fold in cells expressing SUMO-deficient PR versus WT PR expressing cells. These PR gene signatures were uploaded into Oncomine Research Premium Edition software (Compendia Bioscience, Ann Arbor, MI, USA [37]) and the database was searched for associated concepts.

## Results

### PR SUMOylation alters promoter selection in T47D breast cancer cells

For unknown reasons, there is little overlap between PR-regulated genes in normal, relative to neoplastic, breast tissues [38]. One mechanism for the apparent divergence of PR functions may relate to early events in breast cancer development, such as altered signal transduction. Based in part on our prior studies [13,16,39], we predict that the balance between SUMOylated and phosphorylated (that is, deSUMOylated) PRs is frequently altered in breast cancer, resulting in changes in PR promoter selectivity and altered patterns of gene expression. In a screen of ten breast tumors clinically defined as PR+, we detected a wide range of total PR mRNA (not shown) and protein expression (Figure 1A). Of the seven (out of 10) breast tumors that were confirmed to be PR+ by both RT-qPCR and western blotting, at least five samples (lanes one, three, six, eight, and nine) also clearly contained some level of phospho-Ser294 PR-B (Figure 1A). Remarkably, two of ten tumors (lanes one and three) contained abundant phospho-Ser294 PR-B. Notably, PR-B, but not PR-A, Ser294 is rapidly phosphorylated in response to either progestins or peptide growth factors that input to proline-directed protein kinases, primarily within the MAPK and CDK families [12]. Consistent with this finding, EGF blocked progestin-induced PR-B, but not PR-A SUMOylation [13].

The broad range of PR expression in clinical specimens (Figure 1A and [40]) suggests that PR-dependent gene expression may provide a more accurate marker of PR contribution to breast cancer phenotypes. To address the unique actions of phosphorylated and SUMO-deficient PR-B, we measured the transcriptional profiles of breast cancer cells stably expressing either wild-type (capable of SUMOylation) or SUMO-deficient (K388R mutant/phospho mimic) PR-B molecules using whole genome expression profiling. We first engineered multiple clones of vector-matched PR-null T47D breast cancer cells expressing either WT PR-B or mutant K388R (KR) PR-B that is unable to undergo SUMO modification at Lys388; this SUMO-deficient receptor is a functional mimic for PR-B that is persistently phosphorylated on Ser294 [13,41]. Phospho-Ser294 and S294D receptors are hyperactive transcription factors that undergo rapid ligand-dependent (ubiquitin-mediated) downregulation relative to WT PRs [39]. Cells expressing either WT or KR PR-B were then treated with the synthetic progestin, R5020 (10^-8 ^M), for six hours (Figure 1B). Upon ligand-binding, PR is globally phosphorylated at multiple sites, as indicated by a slight gel upshift [42]. Consistent with our previous reports [13,16] hyperactivated KR PR undergoes slightly more rapid ligand-induced (ubiquitin proteasome-dependent) downregulation (apparent at six hours) relative to WT PR [41]. Using these experimental conditions, global gene expression profiles were simultaneously measured using Illumina HT-12v4 whole genome gene expression bead arrays (Figure 1C). Top regulated genes were organized by heat maps showing up- or down-regulated genes (fold change > 8.0 in at least one sample, BH adjusted *P *< 0.001, Figure 1C). Upon progestin treatment, these cells displayed diverse expression patterns; multiple PR-regulated gene sets became readily apparent (Figure 1C; compare groups of PR-regulated genes upregulated (1a) or downregulated (1b) by LD PRs relative to untreated controls, genes upregulated (2a) or downregulated (2b) by LI PRs relative to PR-null controls, and LD genes upregulated primarily in KR relative to WT (3) or WT relative to KR (4) expressing cell lines).

We identified genes that were upregulated > 1.5 fold by PR in a LD orLI manner and discovered gene expression overlap between cells expressing either KR or WT receptors, as well as subsets of uniquely regulated genes (Figure 1D-E, Additional file 5). We next validated the expression profiles for numerous PR target genes from these classes using RT-qPCR (Figure 1F). Notably, *RGS2 *expression (primarily upregulated by the KR receptor) is over-expressed in the basal/myoepithelial compartment and substantially elevated in a majority of breast tumors [43]. In contrast, *BCL2L11 *(BIM) is a pro-apoptotic mediator involved in ERBB/MAPK-dependent luminal cell clearing [44] whose expression is primarily upregulated by WT but not KR receptors. As these examples suggest, our gene array robustly identified diverse classes of PR target genes, and contains gene expression profiles indicative of mechanisms of PR-mediated cellular proliferation and survival.

These results essentially repeated in T47D cells engineered to express either WT or KR PR from an inducible vector system [See Additional file 3]. In this model, inducible expression of PRs (iWT or iKR) is solely dependent on the presence of a small molecule dimerizer, AP21967, added to the cell culture medium; equal levels of either iWT or iKR were induced upon treatment with AP21967 and these receptors were equally phosphorylated on Ser294 in response to progestin [See Additional file 2]. Cells were treated with AP21967 (10^-9 ^M) and R5020 (10^-8 ^M) and assayed for changes in gene expression using the Affymetrix U133A 2.0 microarray platform. Remarkably, PR-dependent gene expression profiles obtained from T47D cells stably expressing PR (assayed using the Illumina platform) were significantly similar to gene array data obtained from the same parental cells (T47D) inducibly expressing PR (assayed via the Affymetrix platform; see Additional file 2). Together, our arrays identified a greater number of PR regulated genes (> 1.5 fold, BH adjusted *P *< 0.01) than previous reports [21,22]; microarray platforms now contain thousands more 'reporters' relative to earlier technologies. Notably, we identified 70% of the previously known PR target genes but also revealed hundreds of novel PR target genes [See Additional file 6].

### Phosphorylation of PR Ser294 drives SUMO-deficient PR gene expression

To investigate mechanisms of regulation of 'SUMO-sensitive' PR-target genes, we selected four genes (*MSX2*, *RGS2*, *MAP1A *and *PDK4*) from our microarray analysis for further study. These specific genes were dramatically upregulated in cells expressing KR, but not WT receptors (Figure 1D, yellow category). A query of the Oncomine database [36] demonstrated that all four genes are amplified in breast carcinomas relative to normal breast tissue and blood (Figure 2A). To validate SUMO-dependent changes in PR target gene expression in an additional breast cancer model, we stably introduced vector control, WT or KR receptors into MCF-7 cells expressing low levels of endogenous PR (in the absence of estrogen). These cells were treated with vehicle control (ethanol) or R5020 (10^-8 ^M) in the absence or presence of the PR antagonist, RU486 (10^-7 ^M) for six hours (Figure 2B). Notably, progestin-induced gene expression profiles in MCF-7 cells were nearly identical to those obtained in our T47D cell models (*MSX2*, *RGS2*, *MAP1A*, and *PDK4*). Additionally, their R5020-induced mRNA expression was completely abolished by addition of RU486, indicating that regulation of these genes is indeed entirely PR-dependent.

We showed previously that SUMO-deficient KR receptors closely mimic phospho-Ser294 (WT) PR species [13]. To demonstrate the phosphorylation-dependence of PR regulation on the same set of genes (*MSX2*, *RGS2*, *MAP1A*, and *PDK4*), we employed PR-null T47D cells or T47D cells stably expressing WT, KR, or phospho-mutant S294A (SA) PR-B [41]. Mutation of PR Ser294 results in a heavily SUMOylated receptor that is transcriptionally repressive, as measured by luciferase reporter assays [13]. Consistent with this finding, progestin-induced upregulation of endogenous PR target genes was blocked in cells expressing S294A PR relative to cells expressing SUMO-deficient KR PR (Figure 2C). Furthermore, progestin-induced gene expression was rescued (that is, comparable to that induced in R5020-treated KR cells) in cells expressing the PR double mutant (KRSA), containing point mutations at both Ser294 and Lys388, suggesting that PR deSUMOylation is the dominant event required for LD upregulation (derepression) of these phosphorylation-dependent PR target genes.

Treatment of breast cancer cells with EGF induces robust PR Ser294 phosphorylation and deSUMOylation [13]. We therefore pre-treated T47D cells stably expressing WT PR with EGF (100 ng/ml) followed by vehicle control or R5020 (10^-8 ^M). Both *MAP1A *and *RGS2 *were insensitive to EGF alone over a two-day time course (Figure 2D). However, EGF pre-treatment significantly augmented progestin stimulated mRNA expression of both genes (Figure 2D). We observed similar results for *RGS2*, but not *MAP1A *expression in parental (expressing both endogenous PR-A and PR-B isoforms) T47Dco cells treated for six hours (Figure 2E). Perhaps not surprisingly, multiple factors (that is, strength and duration of PR phosphorylation, transcriptional activity, and protein levels) likely influence the kinetics of PR-regulated *MAP1A *expression in cells stimulated broadly with growth factors. Notably, in T47D cells stably expressing WT PR-B, *MAP1A *mRNA expression was synergistically upregulated following just three hour of treatment with progestin plus heregulin-β1; progestin-alone approached this by 24 hours (data not shown). Taken together, our data suggest that PR dynamically regulates multiple endogenous genes according to its phosphorylation and SUMOylation status; growth factors favor phospho-PR that act as derepressed transcription factors.

### PR SUMO modification provides a mechanism for promoter selection

**Figure 3:**
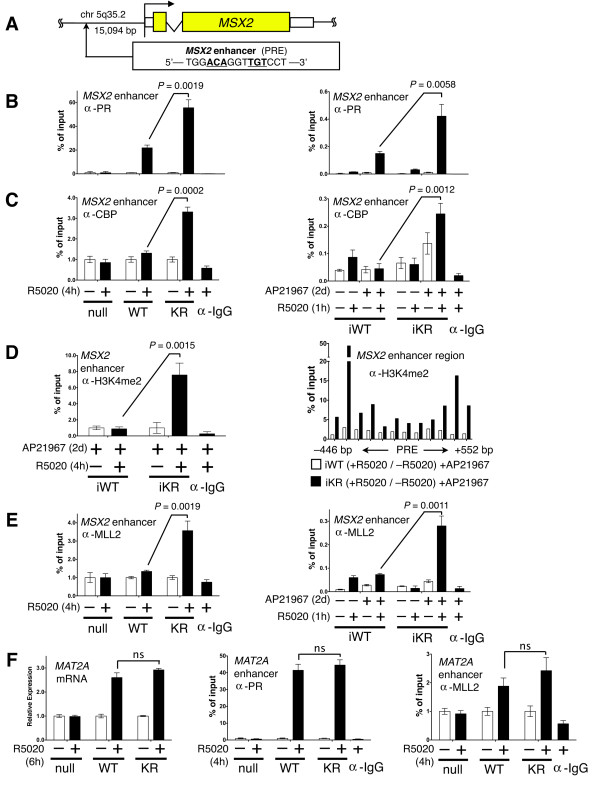
**Promoter selectivity is achieved through increased recruitment of SUMO-deficient KR PR, CBP, MLL2 and histone tail modification, H3K4me2, to enhancer loci**. (**A**) Schematic showing the *MSX2 *gene PRE-containing enhancer region located 15,094 bp upstream from the transcriptional start site. (**B**) Relative recruitment of PR to the *MSX2 *enhancer region was measured by ChIP-qPCR assays in T47D cells expressing constitutive PR null, WT or KR PR after treatment with R5020 for one or four hours. PR recruitment values were normalized as a percentage of input chromatin DNA values. To control for background non-specific antibody binding, immunoprecipitated chromatin contained a mixture from all samples with an IgG antibody. Similar ChIP results were obtained in T47D cells expressing inducible PR (right side). (**C**) The relative recruitment of CBP to the *MSX2 *enhancer region was measured as described in part *B*. (**D**) Levels of H3K4 dimethylation at the *MSX2 *enhancer were measured in the inducible PR expressing cell lines (iWT and iKR). The presence of H3K4me2 was determined at the *MSX2 *enhancer, up/downstream from the PRE, using overlapping qPCR products that span the region. (**E**) MLL2 recruitment to the *MSX2 *enhancer region was determined in T47D cells expressing both constitutive PR and inducible PR, as described in part *B*. (**F**) *MAT2A *gene expression was measured by RT-qPCR in T47D cells expressing stable WT or SUMO-deficient KR PR. Additionally, PR and MLL2 recruitment was quantified in these cells, as measured by standard ChIP-qPCR assay. Data are represented as mean of *n *= 3 +/- SD and significance calculated using Student's t-test. [See also Additional files 7, 5. KR, K388R PR-B mutant]. CBP, CREB-(cAMP-response element-binding protein)-binding protein; ChIP, chromatin immunoprecipitation; H3K4me2, histone H3 lysine 4 dimethylation; IgG, immunoglobulin G; KR, K388R PR-B mutant; MLL2, mixed lineage leukemia 2; PR, progesterone receptor; PRE, progesterone receptor response element; SD, standard deviation; SUMO, small ubiquitin-like modifier; WT, wild type.

Our gene array analyses indicated that SUMO modification of PR alters the magnitude of transcriptional response on selected promoters, while the regulation of other PR target genes is completely insensitive to PR SUMOylation (Figure 1). To investigate mechanisms of PR promoter selection, we examined the recruitment of PR and selected coregulators to the chromatin of differentially regulated PR target genes. We initially focused on *MSX2*. Similar to PR-B, this homeobox transcription factor is essential for mammary gland development and transgenic expression of *MSX2 *causes ductal hyperplasia in mice [45,46]. Functional studies indicate that *MSX2 *induces cyclin D1 and E1 expression [47], is involved in RAS-mediated cellular transformation [48] and drives epithelial-to-mesenchymal transition through downregulation of epithelial markers [49]. Lanigan *et al. *[50] showed that *MSX2 *expression is significantly elevated in both luminal B and HER2-enriched molecular subtypes of breast cancer, despite being associated with good prognosis (that is, similar to ER and PR). We identified multiple consensus progesterone response element (PRE) sequences up- and downstream of the *MSX2 *transcriptional start site using MatInspector software [51]. In particular, one PRE aligned with a region of known PR recruitment, based on the PR cistrome (derived from unpublished ChIP-chip experiments kindly provided by Myles Brown, Harvard). Recall that *MSX2 *is transcriptionally upregulated in response to progestin treatment of T47D or MCF-7 cells stably or inducibly (T47D) expressing SUMO-deficient PR, but not WT receptors (Figure 2B-C, Additional file 3). To investigate direct recruitment of PR to the PRE enhancer region of *MSX2 *(Figure 3A), we treated cells constitutively (or inducibly) expressing either WT or KR PR with R5020 (10^-8 ^M), and performed ChIP assays. Following progestin treatment, both WT and KR PR were readily detected at the PRE enhancer region (Figure 3B left), although we detected no transcriptional activity (mRNA levels as measured by RT-qPCR) in progestin-treated cells expressing WT PR (Figure 2B-C, Additional file 3). Notably, significantly more SUMO-deficient KR PR was recruited to the *MSX2 *enhancer locus relative to that of WT PR. This finding repeated in cells expressing inducible PR (Figure 3B right) as well as at PRE-containing enhancers of multiple other genes upregulated by SUMO-deficient PR [See Additional file 7]. We then investigated the recruitment of a common PR transcriptional coactivator, cAMP-response element-binding protein (CREB)-binding protein (CBP) to the *MSX2 *enhancer locus. CBP interacts with multiple nuclear receptors, functions as a transcriptional scaffold, and has histone acetyltransferase (HAT) activity [52-54]. Using ChIP assays, we determined that upon progestin treatment, CBP recruitment to the *MSX2 *locus is significantly elevated in cells expressing SUMO-deficient KR PR, but not WT PR (Figure 3C). Consistent with the increased presence of this coactivator associated with KR PR, we observed increased recruitment of total and functionally active phospho-Ser5 RNA polymerase II to the *MSX2 *proximal promoter region in progestin-treated cells expressing iKR PR relative to cells expressing iWT PR [See Additional file 8]. These data may explain why, although WT PR is clearly recruited to this region in the presence of progestin (Figure 3B), significant mRNA expression does not occur (Figure 2B-C, Additional file 3). We previously reported constitutive association of deSUMOylated PRs and steroid receptor coactivator 1 (SRC1) at endogenous gene loci [16].

Histone tail modifications (methylation, acetylation, phosphorylation, and so on) are epigenetic modifications known to significantly impact chromatin dynamics and thereby affect changes in gene expression (reviewed in [55]). Generally, histone H3 Lys4 dimethylation (H3K4me2) is an epigenetic mark associated with transcriptional activation [56,57]. H3K4me2 marks areas of transcription factor-facilitated paired nucleosome positioning, and is an indicator of nearby gene activation [57]. To measure the level of H3K4me2 at the *MSX2 *enhancer locus, T47D cells expressing inducible PRs (iWT and iKR) were treated with R5020 (10^-8 ^M) for four hours and nucleosomes were isolated after micrococcal nuclease (MNase) digestion; histone methylation was determined by ChIP, followed by qPCR (Figure 3D left). H3K4me2 levels were elevated in progestin-treated cells expressing iKR relative to cells expressing iWT PR. We also measured the R5020-induced fold change in H3K4me2 surrounding the *MSX2 *PRE locus (approximately 500 bp up- and downstream using overlapping qPCR products) to visualize local histone dimethylation patterns (Figure 3D right). Progestin-dependent H3K4me2 was enriched in cells expressing SUMO-deficient iKR PR compared to cells expressing iWT. Indeed, the higher levels of histone methylation flanking the PRE sequence are likely a consequence of nucleosome remodeling and spreading that facilitates recruitment of transcription factor complexes at this functional enhancer region [57].

These results suggest that one or more histone methyltransferases are differentially recruited to the *MSX2 *enhancer in cells expressing either iWT or iKR PR. Recently, a chromatin remodeling complex, including the subunit mixed lineage leukemia 2 (MLL2) methyltransferase, was implicated in progestin-dependent H3K4 trimethylation [58]. Additionally, ER-alpha interacts directly with MLL2 though its LXXLL motifs and MLL2 mediates estrogen-dependent transcriptional upregulation in MCF-7 cells [59]. Using both stable and inducible T47D models, we discovered that MLL2 is significantly recruited to the *MSX2 *enhancer in progestin treated cells expressing SUMO-deficient KR PR, but not WT PR (Figure 3E).

Finally, we measured the relative recruitment of PR to a PRE-containing enhancer locus near *MAT2A*, a control PR-target gene that is insensitive to PR SUMOylation status (Figure 1D, green category). *MAT2A *mRNA expression was equally upregulated in progestin-treated cells expressing either WT or KR PR (Figure 3F left). Likewise, progestin-dependent recruitment of PR and MLL2 to the same PRE-containing region in the *MAT2A *enhancer was very similar in cells expressing either WT or KR PR (Figure 3F center and right). Taken together, these data suggest that enhancer/promoter structure (in chromatin) functions in combination with PR SUMOylation to block important interactions between PR and mediators of early chromatin remodeling (MLL2) as well as major coregulators, including CBP; higher levels of these factors were specifically associated with 'sensitive' PRE regions in cells expressing SUMO-deficient PR. Perhaps SUMO-sensitive enhancer regions require PR-dependent recruitment of MLL2 in order to initiate changes in nucleosome positioning at relatively 'closed' regions (that is, with regard to genes like *MSX2*). In contrast, pre-existing 'open' regions may be insensitive to PR SUMO modification (that is, with regard to genes like *MAT2A*). Additionally, preferential association of SUMO-deficient PR with other factors (that is, pioneer-type transcription factors) may contribute to PR promoter selection; KR recruitment to the *MSX2 *enhancer region is significantly enhanced relative to WT receptor in the presence of progestin (Figure 3B). These questions await further detailed global gene and cistrome analyses (see Discussion).

### SUMO-deficient phospho-PR promote increased cell proliferation and decreased apoptosis

**Figure 4:**
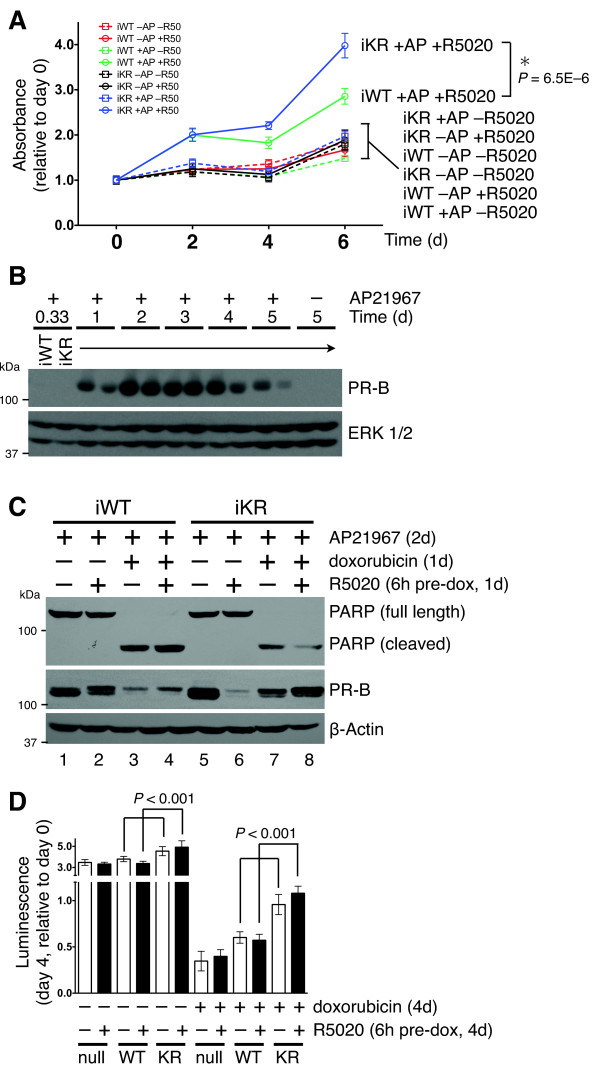
**SUMO-deficient progesterone receptors promote increased cell proliferation and decreased apoptosis**. (**A**) The proliferative potential of T47D cell lines expressing inducible PR was measured using MTT assays in the presence of progestin (R5020) and inducer, AP21967 (AP) (**B**) Western blot showing that inducible PR expression is sustained for at least five days following the addition of AP21967 to the cell culture media, ERK1/2 western blotting was performed as a loading control. (**C**) Apoptosis occurring in cells expressing inducible PRs was detected by western blotting for poly (ADP)-ribose polymerase 1 (PARP) cleavage. Cells were treated with progestin and/or doxorubicin before protein harvest. (**D**) Proliferation and apoptosis was measured in cells constitutively expressing PR using cell viability luciferase assays, where day 4 luminescence was normalized to day 0. Pooled data are represented as mean of *n *= 6 +/- SD and significance calculated using Student's t-test. MTT, 3-(4,5-dimethylthiazol-2-yl)-2,5-diphenyltetrazolium bromide; n, number; PR, progesterone receptor; SD, standard deviation; SUMO, small ubiquitin-like modifier.

Ingenuity Pathway Analysis (IPA, Ingenuity Systems, [60]) software contains a large database of genes that are manually assigned to molecularly defined pathways, biological functions or disease states, and based on current literature. Using this tool, we compared ligand-dependent upregulated genes (> 2 fold, BH adjusted *P *< 0.01) in cells stably expressing either WT or KR receptors. Upon progestin treatment, SUMO-deficient PR, but not WT, significantly upregulated gene sets assigned to multiple proliferative and pro-survival biological functions [See Additional file 2]. We showed that breast cancer cells stably expressing SUMO-deficient PR exhibit increased growth in soft-agar relative to cells stably expressing either WT or phosphorylation-deficient S294A PR [13,16]. We performed MTT proliferation assays using our inducible models (Figure 4A). The advantage of this isogenic system is the elimination of clonal variation in cell growth/death rates and phenotypic drift that can occur in stable cell line models. Cells were plated at equal density on day zero and treated with or without the AP21967 compound to induce PR expression, prior to exposure to either vehicle (ethanol) or progestin (R5020). Progestin-treated cells expressing iWT or iKR PRs grew faster than their un-induced or untreated counterparts. However, by day six of continuous exposure to both AP21967 and R5020, significantly more cells were present in cultures expressing iKR relative to those expressing iWT receptors, while all control groups remained very similar. Western blotting demonstrated that inducible PR expression was sustained when AP21967 was added to the cell culture media and that comparable levels of iWT and iKR PR protein were expressed (Figure 4B).

MTT assays measure viable (surviving) cells over time and PRs have been implicated in breast cancer cell pro-survival [7,61]. Thus, we also measured cleavage of PARP as an indirect indicator of apoptosis. PARP is targeted for cleavage at Asp214 by activated caspase-3 and is a sensitive measure of committed apoptotic signaling [62]. PR expression was induced by AP21967 treatment and cells were pre-treated with R5020 for six hours to activate the respective iWT or SUMO-deficient iKR gene expression programs. Following R5020 pre-treatment, doxorubicin was added to the cell culture medium to induce apoptosis for one day, after which the cell lysate was harvested and the relative levels of cleaved PARP were measured by western blotting (Figure 4C). Notably, doxorubicin-treated cells expressing SUMO-deficient iKR PR had reduced levels of PARP cleavage relative to cells expressing iWT PR, especially in cells pre-treated with R5020 (compare lanes four and eight). Doxorubicin treatment reduced both WT and KR PR protein expression (Figure 4C, compare lanes one and three, or lanes five and seven). However, in multiple repeat experiments normalized to protein expression changes, cells expressing iKR PR consistently exhibited reduced PARP cleavage relative to cells expressing iWT PR. These findings were validated in T47D cells stably expressing PRs. PR-null cells and cells stably expressing either WT or KR PR were plated in complete media, serum starved and treated with R5020, with or without doxorubicin (Figure 4D). Again, we observed significantly increased cell viability in progestin-treated cells expressing SUMO-deficient KR PR. Interestingly, when these cells were challenged with cytotoxic concentrations of doxorubicin, their viability was doubled relative to cells expressing WT PR (Figure 4D). These data suggest that SUMO-deficient PRs are important mediators of increased cell proliferation and pro-survival signaling; cells expressing modified PRs undergo biological processes consistent with their associated gene expression profiles (Figure 1).

### The SUMO-deficient PR gene signature is associated with ERBB2 positive breast cancers

Human breast cancers often contain high levels of MAPK, AKT, and/or CDK protein and/or kinase activities, thus favoring PR derepression [13,16]. To probe published human breast cancer databases for evidence of genetic patterns suggestive of phospho-PR-driven (SUMO-deficient) lesions, we first defined unique PR gene signatures comprised of genes whose expression was greater in cells expressing KR relative to cells expressing WT receptors (expression > 1.5 fold in KR versus WT, BH adjusted *P *< 0.01). These genes were predominantly upregulated in cells expressing KR receptors and/or downregulated only in cells expressing WT receptors. This analysis was performed for both ligand-dependent and ligand-independent PR target genes. Using these criteria, unique 151- and 92-gene signatures were created and defined as PR-target genes differentially upregulated (compared to WT) by LD and LI KR receptors, respectively [See Additional file 9].

**Figure 5:**
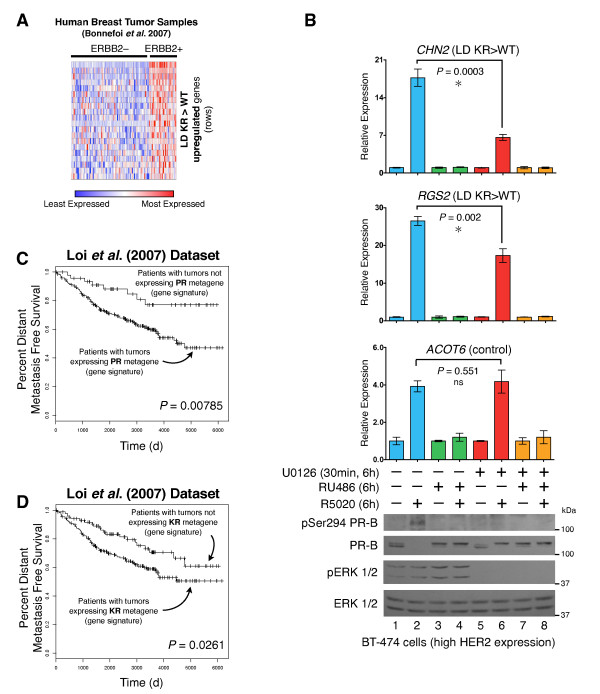
**The SUMO-deficient PR gene expression signature is associated with HER2-positive human breast tumors and predicts reduced patient survival**. (**A**) Normalized gene expression levels (for genes in our LD KR > WT gene signature) are presented for each tumor in the patient cohort [64], organized by ERBB2 status. (**B**) Gene expression levels were measured by RT-qPCR for *CHN2 *and *RGS2 *(both upregulated by SUMO-deficient PR, and members of the LD KR > WT gene signature) and the control gene *ACOT6 *(equally upregulated by both WT and KR receptors) in BT-474 human breast cancer cells. Cells were pre-treated with MEK kinase inhibitor U0126 prior to progestin or antiprogestin co-treatment. Protein levels were evaluated by western blotting for total PR, PR Ser294 phosphorylation, total ERK1/2, and ERK1/2 phosphorylation. (**C**) Kaplan-Meier survival curve for distant metastasis free survival for patients whose tumors expressed the combined T47D metagenes (WT or KR, -/+R5020) relative to patient tumors lacking these metagenes. Patient samples include untreated and tamoxifen-treated ER-positive tumors from the Loi *et al. *dataset [29]. (**D**) Survival curves as in part *C *for patients whose tumors expressed the combined T47D metagenes (KR -R5020, or KR +R5020) relative to patient tumors lacking these metagenes. [See also Additional files 4 and 9]. ER, estrogen receptor; KR, K388R PR-B mutant; LD, ligand dependent; SUMO, small ubiquitin-like modifier; WT, wild type.

These gene signatures were then uploaded into the Oncomine Research Premium Edition (Compendia Bioscience [37]) and the database was interrogated for associated concepts (reviewed in [63]). Oncomine concepts are gene lists defined by specific criteria (for example, top over-expressed genes in a particular tumor cohort). The LD 151-gene signature was associated with multiple breast cancer concepts with high significance (*P *< 0.0001, FDR < 0.01) [See Additional file 8]. Remarkably, five distinct ERBB2-positive breast cancer concepts (two from cell lines and three from tumor cohorts) were independently associated with this LD PR-gene signature. Thus, genes specifically upregulated in the presence of progestin in cells expressing SUMO-deficient PR are among the same genes highly over-expressed (top 5% to 10%) in ERBB2-positive breast cancers (Figure 5A, Additional file 4 Table s1, shaded rows). Notably, the LI 92-gene signature was also significantly associated with at least one ERBB2-positive concept [64]. These data indicate that both LD and LI PR-regulated gene sets are significantly upregulated in protein-kinase-driven tumors, including those known to be ERBB2-positive (Figure 5A).

Expression of these related genetic programs (SUMO-deficient PR and ERBB2 signaling) might represent independent means utilized by breast cancer cells to drive cell proliferation and survival. Indeed, HER2-enriched breast cancers are frequently SR negative [65,66]. Alternatively, these statistically significantly associated concepts may be functionally linked. Luminal breast cancers are primarily SR-positive, but approximately 7% of luminal A and 20% of luminal B tumors are HER2-enriched [67,68]. We therefore tested the PR- and MAPK-dependent regulation of selected genes co-associated with ERBB2 overexpression (Figure 5A) and SUMO-sensitivity (above) in HER2-amplified but SR-positive BT-474 breast cancer cells that contain constitutively activated MAPKs [69]. Antiprogestin treatment dramatically inhibits BT-474 tumor growth in xenograft models [70] and significantly blocks BT-474 cell proliferation in MTT assays conducted over six days *in vitro*; similar results were observed with the MEK inhibitor, U0126 (data not shown). We first measured the expression of PR target genes (*CHN2 *and *RGS2*) primarily regulated by KR (and ERBB2-associated; see Figure 5A rows) but not WT PR, relative to a control gene not sensitive to PR SUMOylation (*ACOT6*; upregulated equally by WT and SUMO-deficient PR, Figure 1F). Remarkably, progestin treatment induced elevated PR-B Ser294 phosphorylation (lane two) and robust upregulation of both *CHN2 *and *RGS2 *in BT-474 cells: 17-fold and 26-fold, respectively (Figure 5B). Recall that *RGS2 *expression is weakly sensitive to progestin treatment in T47D cells expressing WT PR (approximately two-fold) compared to KR PR (approximately 20-fold) (Figure 1F). *ACOT6 *expression was also induced by R5020; expression of all three genes was entirely blocked by antiprogestin RU486 (Figure 5B). Note that when *CHN2 *and *RGS2 *mRNA expression is highest (+R5020; compare lanes one and two), although phospho-Ser294 PR is readily detected, total PR levels are greatly diminished and appear undetectable (lane two), presumably due to LD (proteasome-mediated) downregulation of activated PR species [41]. Pre-treatment of these cells with the MEK kinase inhibitor, U0126, blocked R5020-induced PR Ser294 phosphorylation and partially, but significantly, diminished both *CHN2 *and *RGS2 *expression (Figure 5B, lane six). In contrast, the expression of *ACOT6*, a control gene unaffected by PR SUMO-status, was completely insensitive to MEK kinase inhibition. These data support our hypothesis and demonstrate that phosphorylation events contribute to both expression of the SUMO-deficient PR gene signature and PR-induced proliferation in otherwise unmodified (that is, containing WT PRs) SR-positive breast cancer cells. Similar to *CHN2 *and *RGS2 *(Figure 5B), we predict that a significant number of genes upregulated in ERBB2 overexpressing luminal breast cancers are indeed PR-driven.

The above findings prompted us to test whether PR gene signatures derived from our cell line models were predictive of tumor behavior and patient survival in published human breast tumor cohorts. For example, the Loi *et al. *dataset [29] represents one of the largest collections of survival data from patients whose breast tumors were initially ER+/PR+. Metagenes [71] were isolated from our T47D microarray dataset representing each sample (PR-null, WT PR, KR PR; with or without R5020 treatment). Using Kaplan-Meier survival analysis, we first compared patient tumors that express PR-related metagenes (WT or KR, -/+R5020) to all other patient tumors. This analysis revealed that patients in this tumor cohort whose tumors expressed any PR gene signature (that is, indicative of transcriptionally active PRs) experienced significantly reduced distant metastasis free survival (*P *= 0.000785; Figure 5C). Notably, patient tumors that did not express a PR-related metagene (Figure 5C, top curve) were associated with approximately 80% long-term survival. Presumably, tumors in this group expressed abundant PR, but these receptors remained relatively inactive. Consistent with this notion, high PR mRNA levels were associated with good outcome [29]. Our findings suggest that classification of tumors based on PR expression (rather then activity) is misleading. Interestingly, patients whose tumor gene signature resembled that of T47D cells expressing KR +R5020 trended toward poorer outcome (*P *< 0.1). To include the contribution of LI (KR) PR target genes, we combined patients whose tumors expressed both KR metagenes (KR -R5020 or KR +R5020). These patients experienced significantly reduced distant metastasis free survival relative to those whose tumors did not express either of the two KR metagenes (*P *= 0.0261) (Figure 5D). With respect to node and grade, there was no apparent association with expression of the metagenes. These data suggest that PR-dependent transcription, and in particular, the actions of the deSUMOylated (phospho-Ser294) receptor, contribute to rapid tumor progression and poor outcome in a subset of (luminal) breast cancer patients.

## Discussion

In this study, we performed gene expression profiling to understand better how PR SUMO modification impacts transcriptional activity and promoter selection. Using newly engineered breast cancer cell line models, we identified a (deSUMOylated) PR-driven gene signature that is present in human tumors and associated with decreased patient survival. Previously, we showed that PR phosphorylation at Ser294 antagonizes PR SUMOylation at Lys388 [13]. Our novel data suggest that breast cancer cells may utilize this mechanism to shift PR transcriptional action toward target genes that drive cell proliferation and pro-survival pathways (Figures 4 and 5). Using bioinformatics to analyze global gene expression levels (Figure 1), we identified dramatic differences in transcriptional responses between WT and deSUMOylated PRs [See Additional file 5] that were further characterized by ChIP analysis as alterations in promoter/enhancer selectivity (Figure 3, Additional file 7). Additionally, treatment of unmodified breast cancer cells (or cells expressing only WT PR-B) with EGF further implicated PR Ser294 phosphorylation (PR deSUMOylation) in transcriptional derepression of selected PR target genes (Figure 2). Notably, genes specifically upregulated by SUMO-deficient PR (that is, phospho-PR driven) are significantly associated with genes that are highly expressed in ERBB2-positive human breast tumors and cell lines; our studies support a mechanistic link between phosphorylated (deSUMOylated) PR-B-specific transcriptional action and expression of a subset of ERBB2-associated genes (Figure 5). Collectively, our data provide a strong rationale for further study into mechanisms of phospho-PR-dependent regulation of transcription and the potential contribution of this activity to early or rapid breast cancer progression towards endocrine resistance.

### Gene expression analysis identifies SUMOylation-sensitive PR target genes

We previously reported that PR SUMOylation is transcriptionally repressive at a limited number of endogenous gene loci, including *HBEGF*, *IRS1*, and *STC1 *[13,16]; all three gene products are known to contribute to breast cancer cell proliferation [72-74]. In the work reported here, we performed a comprehensive set of experiments to measure the regulation of endogenous PR target genes using current microarray techniques for whole genome expression profiling in T47D cells expressing either WT PR or SUMO-deficient mutant K388R PR (phospho-mimic), treated with or without the synthetic progestin, R5020. Apart from our investigation of the role of reversible PR SUMOylation, this microarray dataset provides an updated well-controlled analysis (using newly created vector matched cell lines) of WT PR-B transcriptional action in response to progestin treatment. Rigorous independent experiments were performed using additional cell lines and novel cell line clones expressing either constitutive (stable) or inducible WT or mutant PRs, and gene expression levels were measured using distinct microarray platforms (Illumina and Affymetrix). Indeed, our analysis confirmed 70% of previously identified PR target genes [21,22] but also uncovered hundreds of novel PR target genes; many of these are LI examples [See Additional file 6}. This dataset will provide a powerful resource for future studies investigating mechanisms of LD and LI PR-mediated transcriptional regulation.

Notably, our comparison of genes regulated by WT versus KR PRs revealed considerable overlap suggesting that the majority of PR regulated genes are relatively insensitive to dynamic modification of PR-B by SUMOylation/deSUMOylation (Figure 1D-E, green and red Venn categories). However, within these categories, many genes displayed intermediate (varied) levels of expression when regulated by either WT or KR PR, suggesting that multiple mechanisms impact PR mediated transcription, in part according to PR SUMOylation status. Conversely, smaller subsets of genes were highly sensitive to the SUMOylation-status of PR (Figure 1D-E, blue, yellow, purple and orange Venn categories). Surprisingly, these subsets included genes that were both up and downregulated by KR PR relative to WT controls, suggesting that SUMOylation of PR-B can be either repressive or activating, depending on the promoter context. For example, while many proliferative genes were increased, a number of known tumor suppressor genes were repressed by deSUMOylated (KR) PR; this is a topic for further study.

Based on our previous studies [13,16], we predicted that phospho-Ser294-PRs (that is, that are primarily deSUMOylated) mediate a shift in gene regulation that profoundly affects cancer cell phenotypes. Thus, our goal in the current study was to identify these genes and understand the mechanism(s) of their differential regulation (by WT and KR PR) using entirely new breast cancer cell models. In cells stably expressing S294A PR, a receptor unable to be phosphorylated on Ser294 and thus heavily SUMOylated [13,41], the expression of selected KR-upregulated genes (for example, *MSX2 *and so on) was entirely blocked; transcriptional upregulation was rescued in cells expressing the PR K388R/S294A double mutant (KRSA; Figure 2C). These data demonstrate that PR SUMO modification dominantly represses transcription at PR target genes that are effectively 'derepressed' in response to phosphorylation events. For example, PR-dependent *MSX2 *and *RGS2 *mRNA expression was greatly augmented upon EGF treatment of cells expressing WT PR (Figure 2D). We conclude that PR phosphorylation and deSUMOylation affects global gene expression patterns by dramatically altering PR transcriptional activity and promoter selectivity in breast cancer cells.

### Mechanisms impacting PR promoter selectivity

Our microarray studies clearly demonstrate that PR SUMO modification alters the expression of a broad range of PR target genes but has no effect on others. Little is known about the mechanisms of promoter selectivity. However, this question has been addressed with regard to other SR family members [75]. SR interactions with chromatin are highly dynamic and occur as a rapid and continuous exchange [76]. Thus, concentrated regions of transcription factor 'binding' (as measured by ChIP) actually reflect a shift in the equilibrium towards increased transcription factor occupancy at that region. Multiple factors may influence this equilibrium, such as SR binding to consensus DNA sequences, participation of coregulatory factors within multi-protein complexes and/or sequestration of SRs to specific cellular locations, as well as histone modifications that regulate chromatin accessibility. Additionally, studies of restriction enzymes have revealed mechanisms that facilitate enzyme binding to consensus sequences up to 1,000 times faster than is possible via diffusion alone, suggesting the existence of ancillary factors that facilitate binding [77]. Similarly, recent work has determined that specific proteins called 'pioneer factors' aid in chromatin remodeling and localization of SR transcription factors to nearby genomic binding sites (enhancers) in developmental tissue or cancer specific settings [78-80].

Modification of protein substrates by the addition of SUMO molecules can influence protein-protein interactions and/or alter protein stability, localization, or transcriptional activity (reviewed in [15]). PR SUMOylation (at Lys388) most frequently represses PR transcriptional activity (but can increase it in a promoter dependent manner; Figure 1F*BCL2L11 *and *DNALI1*), and tends to slow the rate of ligand-dependent PR downregulation via proteasome mediated turnover [13], but does not appreciably alter PR location [81]. Numerous genes in our analyses behaved like *MSX2*; expression was substantially upregulated by SUMO-deficient KR PR, but not WT PR (Figure 2C). Additionally, KR PR occupied the *MSX2 *enhancer two to three times more than the WT receptor (Figure 3B). The finding that increased levels of KR PR are recruited to this locus and associated with increased *MSX2 *mRNA expression, suggests that PR SUMOylation (in the context of SUMO-sensitive enhancer regions and chromatin) alters co-factor interactions that occur at the level of PR DNA binding. Related to this finding, PIAS3, a PR SUMOylation E3 ligase, directly inhibits PR binding to PRE DNA sequences *in vitro *[81]. Thus, PIAS3-mediated SUMO conjugation to WT (but not KR) PR may prevent efficient receptor binding to selected PRE sequences, thus subsequently shifting the equilibrium away from PR occupancy at these loci. How this mechanism might be sequence specific or promoter specific remains to be determined.

Promoter structure is likely to be an important determinant of promoter selection by SUMOylated transcription factors, including PR. Holmstrom *et al. *[82] found that SUMOylated GR requires stable interaction with DNA containing multiple GR binding sites in order to efficiently inhibit transcription. Interestingly, GR SUMOylation also selectively affects the transcriptional induction of linked endogenous genes [82]. Related to this finding, recent chromatin modification mapping studies have revealed that histone H3 Lys4 mono- and dimethylation (H3K4me1/2) at enhancers is associated with transcriptionally active genes [57,83]. Indeed, regions of transcription factor accessibility to DNA response elements were first identified as DNase or MNase hypersensitive sites because these regions were relatively free from occupied nucleosomes [84]. H3K4me2 is believed to be an epigenetic marker at functional enhancers that may recruit additional proteins (pioneer factors) to facilitate nucleosome remodeling and accessibility of the region for transcription factor binding [57]. We have not identified the pioneer factors for PR recruitment, but in this study, we observed elevated H3K4 dimethylation at the *MSX2 *enhancer in cells expressing SUMO-deficient KR PR, compared to WT PR. In this model, deSUMOylated PR may preferentially recruit the histone methyltransferase, MLL2 (that is, to the *MSX2 *enhancer), resulting in sustained H3K4 dimethylation that allows formation of productive transcriptional complexes at active sites that are normally repressed by SUMOylated receptors.

Finally, DNA binding specificity for SRs is also highly dependent on sequence composition. Studies investigating GR demonstrate that single base pair changes in consensus GRE/PRE sequences can dramatically affect receptor binding and cofactor interaction [85]. Thus, DNA itself appears to be a sequence specific allosteric ligand for SRs, which can directly influence promoter selectivity and transcriptional consequences. SUMOylated GRs appear to prefer near-perfect consensus GR-binding sites [82]. Notably, as with PR, site-specific phosphorylation of GR also alters its promoter preference [86]. It is currently unknown whether SUMOylated versus deSUMOylated PRs differentially recognize different PRE sequences (that is, we did not perform ChIP-seq experiments to identify all PR-binding sites). However, this seems plausible because SUMO modifications can dramatically alter substrate protein conformation. Clearly, deSUMOylated PRs are capable of recruiting abundant PR coactivators (CBP, MLL2) to enhancer regions; the more rapid or stable creation of functional transcriptional complexes may account for the increased 'sampling' or use of selected promoters by KR relative to WT PRs (Figure 3). Our analysis revealed no obvious global signal(s) that could account for preferential repression or activation of selected enhancer regions over others by SUMOylated or deSUMOylated PRs. Studies to map the WT and KR PR cistromes are on-going.

### Clinical implications of deSUMOylated PR gene expression

Targeting ER function in luminal breast cancers with selective ER modulators (SERMs, such as tamoxifen) and/or aromatase inhibitors (for example, anastrozole, letrozole, or exemestane) is very effective for a majority of women [87,88]. Indeed, because SR cross-talk with growth factor signaling pathways is extensive and tumors tend to progress towards endocrine resistance under the influence of heightened growth factor signaling, combination therapies targeting both ER and ERBB receptors enhance progression free survival [89,90]. We have uncovered a unique set of genes that were upregulated, or derepressed, by deSUMOylated (phospho-mimic) PR species under both LD (151 genes) and LI conditions (92 genes) [See Additional file 9]. Elevated expression of these genes may signify tumors that are primarily driven by hyperactive phospho-PR (deSUMOylated) species, particularly in cancers characterized by activated growth factor signaling cascades. For example, MAPK and CDK2 or CDK4/6 are known drivers of breast cancer progression that likely induce persistent PR Ser294 phosphorylation in some breast tumors (Figure 1A). We predict that patients with luminal-type (ER+/PR+) breast tumors that express this 'phospho-PR' gene signature exist (see Figure 1A and Additional file 9) and that this subset, if identified early, could benefit from endocrine therapies that include the use of newer highly selective antiprogestins (that is, ZK 230211, CDB-4124), perhaps in combination with currently used antiestrogens and/or growth factor pathway inhibition.

Indeed, much research has shown that PR is not only a clinical marker of functional ER expression, but also an important independent driver of tumor progression (reviewed in [91]). Notably, as SR+ luminal A-type tumors progress towards a more aggressive growth factor-high luminal B-type phenotype, SR expression begins to decline, starting with PR loss. These poor prognosis luminal-B-type tumors are often clinically characterized as ER+/PR-low or null and are more likely to become endocrine resistant. We showed previously that deSUMOylated phospho-PR function as hyperactive receptors but also turnover rapidly via the ubiquitin-proteasome pathway (Figure 1Band [41]). In fact, when PR-dependent transcription peaks, as measured by RT-qPCR of endogenous gene readouts (via mRNA levels, as in Figure 5B) or using reporter genes, PR protein levels are virtually undetectable [39]. This finding raises the important question of whether PR is also hyperactive in a subset of breast tumors that are clinically defined as PR-low or null (that is, as generally measured by methods of total protein detection in clinical settings). Interestingly, breast tumors are capable of *de novo *progesterone synthesis, a process mediated by growth factor-dependent signaling [92-94]. Tumor-cell (local) production of progesterone may contribute to sustained PR action (that is, at LD genes) in more aggressive ER+/PR+ tumors.

Surprisingly, we found that breast cancer cells expressing deSUMOylated phospho-PR drive the expression of cell proliferation genes [See Additional file 9], many directly involved in positive regulation of the ERBB/MAPK signaling pathway, thus setting up a type of 'feed-forward' vicious cycle that is clearly associated with tumor progression [68,95]. Our data suggest that phospho-PR may act as a driver of this transition (that is, tumor progression towards the gain of growth factor-driven pathways that can precede SR loss) as indicated by significant similarity to our uniquely defined PR signatures [See Additional file 9]. Our key findings are supported by available clinical data from the Women's Health Initiative and Million Women's Study showing that breast tumors that arose in women taking a progestin as part of HRT were more frequent, larger, and of higher grade relative to control groups [4,96]. Remarkably, a recent analysis of these data demonstrated that estrogen-only HRT may actually protect women from invasive breast cancer [3,6]. Taken together with the work of others [97-99], our data support the concept that targeting PR action in breast cancer patients may be highly beneficial, especially for patients that become SERM resistant. Of note, roughly 40% of patients will initially fail or eventually develop resistance to endocrine therapies aimed solely at targeting estrogen action; this represents a large and underserved population.

The intense study surrounding the molecular subtypes of breast cancer has provided great insights into genetic characteristics of this heterogeneous cancer [68], but current targeted therapies are still focused on a small number of clinical-pathological markers. While it is true that knowing the status of various markers (for example, ER, PR, and HER2) has prognostic value and can inform current therapies, measuring mRNA levels for an expanded number of relevant genes (that is, gene signatures) will provide more sensitive and specific information regarding the genetic pathways active in the tumor. This knowledge could be used to inform clinical decisions, especially when targeted therapies are considered. Thus, there has been rapid expansion of prognostic mRNA expression based assays to classify breast tumors [29,100-102]. However, currently available prognostic signatures fail to link changes in gene expression to the molecular drivers present in a given tumor. Here, we have identified a PR-dependent gene signature more likely to characterize aggressive tumors (Figure 5D, Additional file 9). Our studies implicate deSUMOylated phospho-PRs as major drivers of this phenotype. Although validation studies in animal models are required (in progress), our studies strongly support the use of antiprogestins as valuable additions to state-of-the-art antiestrogen-based endocrine therapies. Identification of patients with PR-driven tumors (that contain a phospho-PR gene signature) may allow early intervention aimed at preventing the development of endocrine resistance.

## Conclusions

We have determined that PR transcriptional action is more complex than originally thought, insofar as PR are sensors for mitogenic stimuli whereby phosphorylation events drive the receptor toward the deSUMOylated state, resulting in a dramatically altered transcriptional program that promotes cell proliferation and pro-survival. We have uncovered a deSUMOylated phospho-PR gene signature of both known and novel PR target genes that is a marker of hyperactive PR signaling in breast cancer cell models; this signature is indeed also present in a subset of patients with recurrent breast cancer (Figures. 1A and 5D). In future, this unique signature may provide a valuable prognostic measure for identifying patients whose tumors are likely to rapidly progress and/or become endocrine-resistant (that is, to estrogen-based therapies).

## Abbreviations

Bp: base pair; BH: Benjamini and Hochberg; BUS: B upstream segment; CBP: CREB-(cAMP-response element-binding protein)-binding protein; CDK: cyclin dependent kinase; ChIP: chromatin immunoprecipitation; cMEM: complete minimal essential medium; DCC: dextran-coated charcoal treated; EGF: epidermal growth factor; ER: estrogen receptor; ERBB2/HER2: human epidermal growth factor receptor 2; FBS: fetal bovine serum; GR: glucocorticoid receptor; GSEA: gene set enrichment analysis; H3K4me2: histone H3 lysine 4 dimethylation; HAT: histone acetyl-transferase; HRT: hormone replacement therapy; IQR: interquartile range; IPA: ingenuity pathway analysis; KR: K388R PR-B mutant; KRSA: K388R and S294A PR-B mutant; LD: ligand dependent; LI: ligand independent; MAPK: mitogen activated protein kinase; MLL2: mixed lineage leukemia 2; MNase: microsomal nuclease; NEAA: non-essential amino acids; PARP: poly (ADP) ribose polymerase 1; PR: progesterone receptor; PRE: progesterone receptor response element; RIPA: radioimmunoprecipitation assay; RTqPCR: reverse transcriptase quantitative polymerase chain reaction; SA: S294A PR-B mutant; SERM: selective estrogen receptor modulator; SR: steroid receptor; SUMO: small ubiquitin-like modifier; WT: wild-type PR-B.

## Competing interests

The authors declare that they have no competing interests.

## Authors' contributions

TPK and CAL conceived of the study, participated in its design and coordination, and drafted the manuscript. TPK performed all experiments and analyses except where noted below. ARD carried out the western blot in Figure 1A, provided valuable feedback during the project, and revised the manuscript. DF carried out the Illumina gene expression dataset normalization, created the heat map in Figure 1C, contributed to the analysis in Additional file 3 and contributed to the manuscript draft (Materials and methods section). KATS carried out the Affymetrix gene expression dataset normalization and contributed to the manuscript draft (Materials and methods section). KRC and SAWF performed the metagene analysis shown in Figure 5C-D, and provided helpful editorial comments during the draft stage. CAL supervised the entire project. All authors read and approved the final manuscript.

## Supplementary Material

Additional file 1 **Genes differentially regulated by wild-type and SUMO-deficient PR**. Gene names and their normalized expression values from each Venn diagram category (Figure 1D-E) were organized into different Excel worksheets. Multiple biological sample comparisons were performed (for example, genes upregulated by both ligand-dependent WT and KR, green category in Venn diagram); genes with absolute value log_2 _fold change > 0.6 (that is, > 1.5 fold up- or down-regulated) and BH adjusted *P *< 0.01 are presented in the corresponding Excel worksheets. In each worksheet, multiple sample comparisons (log_2 _fold change and BH adjusted *P *value) and the log_2 _normalized intensities are displayed for each gene. Data were sorted based on the highest expression (log_2 _fold change) in the specific sample comparison being presented in each corresponding Excel worksheet (columns highlighted with grey cell background color). If multiple probe sets represented a single gene, the probe set with the highest expression value was used in downstream analyses and other probe sets were removed. Click here for file

Additional file 2 **Creation and validation of isogenic models of inducible PR expression in T47D cells**. (**A**) Clonal inducible cell lines were developed as described in the Materials and methods section and PR protein expression was determined by western blotting after treatment with inducer molecule AP21967 for two days and R5020 for one hour. Progestin-dependent PR phosphorylation was measured using a PR phospho-Ser294 specific antibody. Beta-actin western blotting was performed as a loading control. Short-term treatment with R5020 demonstrated progestin-dependent PR global phosphorylation (as indicated by a slight gel upshift in total PR) and equal levels of ligand-dependent Ser294 phosphorylation. (**B**) Gene set enrichment analysis (GSEA) comparison of whole genome expression profiling data sets derived from two independent model systems and platforms: (i) T47D cells stably expressing WT and mutant KR PRs (-/+R5020) using the Illumina HT-12v4 platform and (ii) T47D cells expressing inducible WT or mutant KR PR (-/+AP21967, -/+R5020) using the Affymetrix U133A 2.0 platform. Genes most upregulated in the Illumina dataset by WT +R5020 (or KR +R5020) appear on the far left (darkest red) and genes most downregulated by WT +R5020 (or KR +R5020) appear on the far right side (darkest blue). Using the GSEA application, Affymetrix genes (black vertical bars) were positioned along the Illumina dataset (from upregulated to downregulated genes) and the statistical enrichment score was determined. All the treatment groups between Affymetrix and Illumina were statistically significant (*P *< 0.001). (**C**) Gene expression levels were validated for two PR target genes (*MSX2 *and *MAP1A*) in T47D cell lines expressing iWT and iKR PR. Cells were treated with AP21967 to induce PR expression and co-treated with RU486 and/or R5020 before RT-qPCR gene expression analysis. Data are represented as mean of *n *= 3 +/- SD. Click here for file

Additional file 3 **Overlapping lists of PR-dependent target genes from previously described gene expression microarrays**. Excel workbook comparing previously known PR target genes [21,22] to novel PR target genes discovered herein. Three different gene lists were compared using Venn diagram analyses: genes upregulated by WT PR, upregulated by SUMO-deficient KR PR, or previously known genes upregulated by WT PR-B. Genes identified herein were upregulated > 1.5 fold BH adjusted *P *< 0.01. Analyses were performed for genes upregulated in response to progestin treatment or genes upregulated in ligand-independent conditions. Analyses of genes downregulated by PR were omitted (few PR target genes were previously known to be downregulated by PR-B). Notably, very few ligand-independent PR target genes have been reported to date [21]. Perhaps not surprisingly, we observed little overlap between these datasets. Click here for file

Additional file 4 **Relative recruitment of WT and SUMO-deficient PR molecules to selected PR target gene enhancers**. (A) Recruitment of PR molecules to consensus PRE sequences in upstream promoter/enhancer regions of *RGS2*, *MAP1A*, and *PDK4 *(following one hour R5020) was measured by standard ChIP assay in inducible models of T47D cells expressing WT (iWT) and KR (iKR) receptors. Recruitment of PR to an intronic region of the *HBB *gene was included as a negative control. (B) ChIP assays were performed as in part **A**, to demonstrate differential PR recruitment to a *RGS2 *enhancer in T47D cells stably expressing either WT or SUMO-deficient (KR) PR. Data are represented as mean of *n *= 3 +/- SD. Click here for file

Additional file 5 **Recruitment of phospho-Ser5 and total-RNA polymerase II to the *MSX2 *proximal promoter region**. (**A**) Recruitment of total RNA polymerase II to the *MSX2 *proximal promoter region (following one hour R5020) was measured by standard ChIP assay in inducible models of T47D cells expressing WT (iWT) and KR (iKR) receptors. (**B**) ChIP assay was performed as in part **A**, using an antibody targeting functionally active RNA polymerase II, as measured by detection of CTD Ser5 phosphorylation. Data are represented as mean of *n *= 3 +/- SD. Click here for file

Additional file 6 **SUMO-deficient PR upregulates genes involved in cell proliferation determined by Ingenuity Pathway Analysis**. Significant expression (y-axis) of multiple cellular functions (x-axis) containing genes upregulated by progestin (log2 fold change > 1.0, BH adjusted P < 0.01; common fold change > 2.0) in cells expressing either WT or KR PR. Biological pathways that contain a significant number of upregulated genes display bars above the horizontal line, representing BH adjusted P < 0.05. Click here for file

Additional file 7 **The ligand-dependent (LD) and ligand-independent (LI) KR > WT gene signatures**. For each gene signature, the gene names, normalized expression values, and the BH adjusted P values are provided for all biological samples and sample comparisons. The LD (151 genes) and LI (92 genes) KR > WT gene signature lists are provided in whole. Click here for file

Additional file 8 **Breast tumor Oncomine concepts associated with PR dependent gene signatures**. This Excel workbook contains detailed data produced from the Oncomine analysis described in Figure 5. All Oncomine breast cancer dataset concepts that were associated with various gene signatures are provided, including the LD KR > WT gene signature. Of these, five ERBB2-positive datasets (three tumor and two cell line) were associated with the LD KR > WT concept (rows shown in yellow background). For one of these significantly associated concepts, a table of the overlapping genes present in both the PR-gene signature concept (LD KR > WT) and the ERBB2-positive associated concept (from the Bonnefoi et al. dataset [64]) is available. Also, the top 20 genes presented in the heat map (Figure 5A) are available (shaded with yellow background), in addition to all other genes from the dataset [64] not shown in the heat map. This Oncomine dataset [64] is defined as 'genes over-expressed in Ductal Breast Carcinoma ERBB2-positive tumors'. Click here for file

Additional file 9 **Detailed antibody and PCR primer set information**. This Excel workbook contains all the antibody information and primers sets used in RT- and ChIP-qPCR assays. Click here for file

## References

[B1] Siegel R Naishadham D Jemal A Cancer statistics, 2012 CA Cancer J Clin 2012 62 10 29 10.3322/caac.20138 22237781

[B2] Chlebowski RT Hendrix SL Langer RD Stefanick ML Gass M Lane D Rodabough RJ Gilligan MA Cyr MG Thomson CA Khandekar J Petrovitch H McTiernan A Influence of estrogen plus progestin on breast cancer and mammography in healthy postmenopausal women: the Women's Health Initiative Randomized Trial JAMA 2003 289 3243 3253 10.1001/jama.289.24.3243 12824205

[B3] LaCroix AZ Chlebowski RT Manson JE Aragaki AK Johnson KC Martin L Margolis KL Stefanick ML Brzyski R Curb JD Howard BV Lewis CE Wactawski-Wende J Health outcomes after stopping conjugated equine estrogens among postmenopausal women with prior hysterectomy: a randomized controlled trial JAMA 2011 305 1305 1314 10.1001/jama.2011.382 21467283 PMC3656722

[B4] Million Women Study Collaborators Breast cancer and hormone-replacement therapy in the Million Women Study Lancet 2003 362 419 427 12927427 10.1016/s0140-6736(03)14065-2

[B5] Chlebowski RT Kuller LH Prentice RL Stefanick ML Manson JE Gass M Aragaki AK Ockene JK Lane DS Sarto GE Rajkovic A Schenken R Hendrix SL Ravdin PM Rohan TE Yasmeen S Anderson G Breast cancer after use of estrogen plus progestin in postmenopausal women N Engl J Med 2009 360 573 587 10.1056/NEJMoa0807684 19196674 PMC3963492

[B6] Anderson GL Chlebowski RT Aragaki AK Kuller LH Manson JE Gass M Bluhm E Connelly S Hubbell FA Lane D Martin L Ockene J Rohan T Schenken R Wactawski-Wende J Conjugated equine oestrogen and breast cancer incidence and mortality in postmenopausal women with hysterectomy: extended follow-up of the Women's Health Initiative randomised placebo-controlled trial Lancet Oncol 2012 13 476 486 10.1016/S1470-2045(12)70075-X 22401913 PMC3412626

[B7] Lange CA Challenges to defining a role for progesterone in breast cancer Steroids 2008 73 914 921 10.1016/j.steroids.2007.12.023 18243264 PMC2481303

[B8] Daniel AR Knutson TP Lange CA Signaling inputs to progesterone receptor gene regulation and promoter selectivity Mol Cell Endocrinol 2009 308 47 52 10.1016/j.mce.2009.01.004 19549591 PMC3924551

[B9] Terry KL De Vivo I Titus-Ernstoff L Sluss PM Cramer DW Genetic variation in the progesterone receptor gene and ovarian cancer risk Am J Epidemiol 2005 161 442 451 10.1093/aje/kwi064 15718480 PMC1380205

[B10] De Vivo I Huggins GS Hankinson SE Lescault PJ Boezen M Colditz GA Hunter DJ A functional polymorphism in the promoter of the progesterone receptor gene associated with endometrial cancer risk Proc Natl Acad Sci USA 2002 99 12263 12268 10.1073/pnas.192172299 12218173 PMC129433

[B11] Pooley KA Healey CS Smith PL Pharoah PD Thompson D Tee L West J Jordan C Easton DF Ponder BA Dunning AM Association of the progesterone receptor gene with breast cancer risk: a single-nucleotide polymorphism tagging approach Cancer Epidemiol Biomarkers Prev 2006 15 675 682 10.1158/1055-9965.EPI-05-0679 16614108

[B12] Clemm DL Sherman L Boonyaratanakornkit V Schrader WT Weigel NL Edwards DP Differential hormone-dependent phosphorylation of progesterone receptor A and B forms revealed by a phosphoserine site-specific monoclonal antibody Mol Endocrinol 2000 14 52 65 10.1210/me.14.1.52 10628747

[B13] Daniel AR Faivre EJ Lange CA Phosphorylation-dependent antagonism of sumoylation derepresses progesterone receptor action in breast cancer cells Mol Endocrinol 2007 21 2890 2906 10.1210/me.2007-0248 17717077

[B14] Melchior F SUMO--nonclassical ubiquitin Annu Rev Cell Dev Biol 2000 16 591 626 10.1146/annurev.cellbio.16.1.591 11031248

[B15] Geiss-Friedlander R Melchior F Concepts in sumoylation: a decade on Nature reviews Molecular cell biology 2007 8 947 956 10.1038/nrm2293 18000527

[B16] Daniel AR Lange CA Protein kinases mediate ligand-independent derepression of sumoylated progesterone receptors in breast cancer cells Proc Natl Acad Sci USA 2009 106 14287 14292 10.1073/pnas.0905118106 19706513 PMC2732858

[B17] Iniguez-Lluhi JA Pearce D A common motif within the negative regulatory regions of multiple factors inhibits their transcriptional synergy Mol Cell Biol 2000 20 6040 6050 10.1128/MCB.20.16.6040-6050.2000 10913186 PMC86080

[B18] Horwitz KB Mockus MB Lessey BA Variant T47D human breast cancer cells with high progesterone-receptor levels despite estrogen and antiestrogen resistance Cell 1982 28 633 642 10.1016/0092-8674(82)90218-5 7200400

[B19] Sartorius CA Groshong SD Miller LA Powell RL Tung L Takimoto GS Horwitz KB New T47D breast cancer cell lines for the independent study of progesterone B- and A-receptors: only antiprogestin-occupied B-receptors are switched to transcriptional agonists by cAMP Cancer Res 1994 54 3868 3877 8033109

[B20] Hagan CR Regan TM Dressing GE Lange CA ck2-dependent phosphorylation of progesterone receptors (PR) on Ser81 regulates PR-B isoform-specific target gene expression in breast cancer cells Mol Cell Biol 2011 31 2439 2452 10.1128/MCB.01246-10 21518957 PMC3133426

[B21] Jacobsen BM Schittone SA Richer JK Horwitz KB Progesterone-independent effects of human progesterone receptors (PRs) in estrogen receptor-positive breast cancer: PR isoform-specific gene regulation and tumor biology Mol Endocrinol 2005 19 574 587 15563544 10.1210/me.2004-0287

[B22] Richer JK Jacobsen BM Manning NG Abel MG Wolf DM Horwitz KB Differential gene regulation by the two progesterone receptor isoforms in human breast cancer cells J Biol Chem 2002 277 5209 5218 10.1074/jbc.M110090200 11717311

[B23] The R software project http://www.R-project.org

[B24] Gentleman RC Carey VJ Bates DM Bolstad B Dettling M Dudoit S Ellis B Gautier L Ge Y Gentry J Hornik K Hothorn T Huber W Iacus S Irizarry R Leisch F Li C Maechler M Rossini AJ Sawitzki G Smith C Smyth G Tierney L Yang JY Zhang J Bioconductor: open software development for computational biology and bioinformatics Genome Biol 2004 5 R80 10.1186/gb-2004-5-10-r80 15461798 PMC545600

[B25] Benjamini Y Hochberg Y Controlling the false discovery rate - a practical and powerful approach to multiple testing J Roy Stat Soc B Met 1995 57 289 300

[B26] Irizarry RA Bolstad BM Collin F Cope LM Hobbs B Speed TP Summaries of Affymetrix GeneChip probe level data Nucleic Acids Res 2003 31 e15 10.1093/nar/gng015 12582260 PMC150247

[B27] Liu WM High density DNA microarrays: algorithms and biomedical applications Curr Med Chem 2004 11 2143 2151 15279554 10.2174/0929867043364739

[B28] Gaujoux R Seoighe C A flexible R package for nonnegative matrix factorization BMC Bioinformatics 2010 11 367 10.1186/1471-2105-11-367 20598126 PMC2912887

[B29] Loi S Haibe-Kains B Desmedt C Lallemand F Tutt AM Gillet C Ellis P Harris A Bergh J Foekens JA Klijn JGM Larsimont D Buyse M Bontempi G Delorenzi M Piccart MJ Sotiriou C Definition of clinically distinct molecular subtypes in estrogen receptor-positive breast carcinomas through genomic grade J Clin Oncol 2007 25 1239 1246 10.1200/JCO.2006.07.1522 17401012

[B30] Covington K Parikh A The Red-R Framework for Integrated Discovery The Red-R Journal 2011 1 http://www.red-r.org/journal/published-articles/1-08082011-red-r-framework-integrated-discovery

[B31] Oliveros JC VENNY. An interactive tool for comparing lists with Venn Diagrams 2007 http://bioinfogpcnbcsices/tools/venny/indexhtml

[B32] Mootha VK Lindgren CM Eriksson KF Subramanian A Sihag S Lehar J Puigserver P Carlsson E Ridderstrale M Laurila E Houstis N Daly MJ Patterson N Mesirov JP Golub TR Tamayo P Spiegelman B Lander ES Hirschhorn JN Altshuler D Groop LC PGC-1alpha-responsive genes involved in oxidative phosphorylation are coordinately downregulated in human diabetes Nat Genet 2003 34 267 273 10.1038/ng1180 12808457

[B33] Subramanian A Tamayo P Mootha VK Mukherjee S Ebert BL Gillette MA Paulovich A Pomeroy SL Golub TR Lander ES Mesirov JP Gene set enrichment analysis: a knowledge-based approach for interpreting genome-wide expression profiles Proc Natl Acad Sci USA 2005 102 15545 15550 10.1073/pnas.0506580102 16199517 PMC1239896

[B34] Verzi MP Shin H He HH Sulahian R Meyer CA Montgomery RK Fleet JC Brown M Liu XS Shivdasani RA Differentiation-specific histone modifications reveal dynamic chromatin interactions and partners for the intestinal transcription factor CDX2 Dev Cell 2010 19 713 726 10.1016/j.devcel.2010.10.006 21074721 PMC3001591

[B35] Crouch SP Kozlowski R Slater KJ Fletcher J The use of ATP bioluminescence as a measure of cell proliferation and cytotoxicity J Immunol Methods 1993 160 81 88 10.1016/0022-1759(93)90011-U 7680699

[B36] The Oncomine database http://oncomine.org

[B37] Oncomine Research Premium Edition software http://oncomine.com

[B38] Graham JD Mote PA Salagame U van Dijk JH Balleine RL Huschtscha LI Reddel RR Clarke CL DNA replication licensing and progenitor numbers are increased by progesterone in normal human breast Endocrinology 2009 150 3318 3326 10.1210/en.2008-1630 19342456 PMC2703536

[B39] Daniel AR Qiu M Faivre EJ Ostrander JH Skildum A Lange CA Linkage of progestin and epidermal growth factor signaling: phosphorylation of progesterone receptors mediates transcriptional hypersensitivity and increased ligand-independent breast cancer cell growth Steroids 2007 72 188 201 10.1016/j.steroids.2006.11.009 17173941 PMC1850618

[B40] Liu S Chia SK Mehl E Leung S Rajput A Cheang MCU Nielsen TO Progesterone receptor is a significant factor associated with clinical outcomes and effect of adjuvant tamoxifen therapy in breast cancer patients Breast Cancer Res Treat 2010 119 53 61 10.1007/s10549-009-0318-0 19205877

[B41] Lange CA Shen T Horwitz KB Phosphorylation of human progesterone receptors at serine-294 by mitogen-activated protein kinase signals their degradation by the 26S proteasome Proc Natl Acad Sci USA 2000 97 1032 1037 10.1073/pnas.97.3.1032 10655479 PMC15511

[B42] Takimoto GS Hovland AR Tasset DM Melville MY Tung L Horwitz KB Role of phosphorylation on DNA binding and transcriptional functions of human progesterone receptors J Biol Chem 1996 271 13308 13316 10.1074/jbc.271.23.13308 8662865

[B43] Smalley MJ Iravani M Leao M Grigoriadis A Kendrick H Dexter T Fenwick K Regan JL Britt K McDonald S Lord CJ Mackay A Ashworth A Regulator of G-protein signalling 2 mRNA is differentially expressed in mammary epithelial subpopulations and over-expressed in the majority of breast cancers Breast Cancer Res 2007 9 R85 10.1186/bcr1834 18067675 PMC2246188

[B44] Reginato MJ Mills KR Becker EB Lynch DK Bonni A Muthuswamy SK Brugge JS Bim regulation of lumen formation in cultured mammary epithelial acini is targeted by oncogenes Mol Cell Biol 2005 25 4591 4601 10.1128/MCB.25.11.4591-4601.2005 15899862 PMC1140636

[B45] Satoh K Hovey RC Malewski T Warri A Goldhar AS Ginsburg E Saito K Lydon JP Vonderhaar BK Progesterone enhances branching morphogenesis in the mouse mammary gland by increased expression of Msx2 Oncogene 2007 26 7526 7534 10.1038/sj.onc.1210555 17546050

[B46] Satokata I Ma L Ohshima H Bei M Woo I Nishizawa K Maeda T Takano Y Uchiyama M Heaney S Peters H Tang Z Maxson R Maas R Msx2 deficiency in mice causes pleiotropic defects in bone growth and ectodermal organ formation Nat Genet 2000 24 391 395 10.1038/74231 10742104

[B47] Satoh K Ginsburg E Vonderhaar BK Msx-1 and Msx-2 in mammary gland development J Mammary Gland Biol Neoplasia 2004 9 195 205 15300013 10.1023/B:JOMG.0000037162.84758.b5

[B48] Takahashi C Akiyama N Kitayama H Takai S Noda M Possible involvement of MSX-2 homeoprotein in v-ras-induced transformation Leukemia 1997 11 Suppl 3 340 343 9209384

[B49] di Bari MG Ginsburg E Plant J Strizzi L Salomon DS Vonderhaar BK Msx2 induces epithelial-mesenchymal transition in mouse mammary epithelial cells through upregulation of Cripto-1 J Cell Physiol 2009 219 659 666 10.1002/jcp.21712 19170109 PMC2753837

[B50] Lanigan F Gremel G Hughes R Brennan DJ Martin F Jirstrom K Gallagher WM Homeobox transcription factor muscle segment homeobox 2 (Msx2) correlates with good prognosis in breast cancer patients and induces apoptosis in vitro Breast Cancer Res 2010 12 R59 10.1186/bcr2621 20682066 PMC2949651

[B51] Cartharius K Frech K Grote K Klocke B Haltmeier M Klingenhoff A Frisch M Bayerlein M Werner T MatInspector and beyond: promoter analysis based on transcription factor binding sites Bioinformatics 2005 21 2933 2942 10.1093/bioinformatics/bti473 15860560

[B52] Li X Wong J Tsai SY Tsai MJ O'Malley BW Progesterone and glucocorticoid receptors recruit distinct coactivator complexes and promote distinct patterns of local chromatin modification Mol Cell Biol 2003 23 3763 3773 10.1128/MCB.23.11.3763-3773.2003 12748280 PMC155204

[B53] Lambert JR Nordeen SK CBP recruitment and histone acetylation in differential gene induction by glucocorticoids and progestins Mol Endocrinol 2003 17 1085 1094 10.1210/me.2001-0183 12637584

[B54] Ogryzko VV Schiltz RL Russanova V Howard BH Nakatani Y The transcriptional coactivators p300 and CBP are histone acetyltransferases Cell 1996 87 953 959 10.1016/S0092-8674(00)82001-2 8945521

[B55] Ong CT Corces VG Enhancer function: new insights into the regulation of tissue-specific gene expression Nat Rev Genet 2011 12 283 293 21358745 10.1038/nrg2957 PMC3175006

[B56] Barski A Cuddapah S Cui K Roh TY Schones DE Wang Z Wei G Chepelev I Zhao K High-resolution profiling of histone methylations in the human genome Cell 2007 129 823 837 10.1016/j.cell.2007.05.009 17512414

[B57] He HH Meyer CA Shin H Bailey ST Wei G Wang Q Zhang Y Xu K Ni M Lupien M Mieczkowski P Lieb JD Zhao K Brown M Liu XS Nucleosome dynamics define transcriptional enhancers Nat Genet 2010 42 343 347 10.1038/ng.545 20208536 PMC2932437

[B58] Vicent GP Nacht AS Font-Mateu J Castellano G Gaveglia L Ballare C Beato M Four enzymes cooperate to displace histone H1 during the first minute of hormonal gene activation Genes Dev 2011 25 845 862 10.1101/gad.621811 21447625 PMC3078709

[B59] Mo R Rao SM Zhu YJ Identification of the MLL2 complex as a coactivator for estrogen receptor alpha J Biol Chem 2006 281 15714 15720 10.1074/jbc.M513245200 16603732

[B60] Ingenuity Pathway Analysis Software http://www.ingenuity.com

[B61] Moore MR Conover JL Franks KM Progestin effects on long-term growth, death, and Bcl-xL in breast cancer cells Biochem Biophys Res Commun 2000 277 650 654 10.1006/bbrc.2000.3728 11062008

[B62] Nicholson DW Ali A Thornberry NA Vaillancourt JP Ding CK Gallant M Gareau Y Griffin PR Labelle M Lazebnik YA Identification and inhibition of the ICE/CED-3 protease necessary for mammalian apoptosis Nature 1995 376 37 43 10.1038/376037a0 7596430

[B63] Rhodes DR Kalyana-Sundaram S Mahavisno V Varambally R Yu J Briggs BB Barrette TR Anstet MJ Kincead-Beal C Kulkarni P Varambally S Ghosh D Chinnaiyan AM Oncomine 3.0: genes, pathways, and networks in a collection of 18,000 cancer gene expression profiles Neoplasia 2007 9 166 180 10.1593/neo.07112 17356713 PMC1813932

[B64] Bonnefoi H Potti A Delorenzi M Mauriac L Campone M Tubiana-Hulin M Petit T Rouanet P Jassem J Blot E Becette V Farmer P Andre S Acharya CR Mukherjee S Cameron D Bergh J Nevins JR Iggo RD Validation of gene signatures that predict the response of breast cancer to neoadjuvant chemotherapy: a substudy of the EORTC 10994/BIG 00-01 clinical trial Lancet Oncol 2007 8 1071 1078 10.1016/S1470-2045(07)70345-5 18024211

[B65] Perou CM Sørlie T Eisen MB van de Rijn M Jeffrey SS Rees CA Pollack JR Ross DT Johnsen H Akslen LA Fluge O Pergamenschikov A Williams C Zhu SX Lønning PE Børresen-Dale AL Brown PO Botstein D Molecular portraits of human breast tumours Nature 2000 406 747 752 10.1038/35021093 10963602

[B66] Sørlie T Perou CM Tibshirani R Aas T Geisler S Johnsen H Hastie T Eisen MB van de Rijn M Jeffrey SS Thorsen T Quist H Matese JC Brown PO Botstein D Eystein Lønning P Børresen-Dale AL Gene expression patterns of breast carcinomas distinguish tumor subclasses with clinical implications Proc Natl Acad Sci USA 2001 98 10869 10874 10.1073/pnas.191367098 11553815 PMC58566

[B67] Cheang MC Chia SK Voduc D Gao D Leung S Snider J Watson M Davies S Bernard PS Parker JS Perou CM Ellis MJ Nielsen TO Ki67 index, HER2 status, and prognosis of patients with luminal B breast cancer J Natl Cancer Inst 2009 101 736 750 10.1093/jnci/djp082 19436038 PMC2684553

[B68] Prat A Perou CM Deconstructing the molecular portraits of breast cancer Mol Oncol 2011 5 5 23 10.1016/j.molonc.2010.11.003 21147047 PMC5528267

[B69] Lenferink AE Busse D Flanagan WM Yakes FM Arteaga CL ErbB2/neu kinase modulates cellular p27(Kip1) and cyclin D1 through multiple signaling pathways Cancer Res 2001 61 6583 6591 11522658

[B70] Liang Y Besch-Williford C Brekken RA Hyder SM Progestin-dependent progression of human breast tumor xenografts: a novel model for evaluating antitumor therapeutics Cancer Res 2007 67 9929 9936 10.1158/0008-5472.CAN-07-1103 17942925

[B71] Huang E Ishida S Pittman J Dressman H Bild A Kloos M D'Amico M Pestell RG West M Nevins JR Gene expression phenotypic models that predict the activity of oncogenic pathways Nat Genet 2003 34 226 230 10.1038/ng1167 12754511

[B72] Beerli RR Hynes NE Epidermal growth factor-related peptides activate distinct subsets of ErbB receptors and differ in their biological activities J Biol Chem 1996 271 6071 6076 10.1074/jbc.271.11.6071 8626392

[B73] Chang AC Jellinek DA Reddel RR Mammalian stanniocalcins and cancer Endocr Relat Cancer 2003 10 359 373 10.1677/erc.0.0100359 14503913

[B74] Byron SA Horwitz KB Richer JK Lange CA Zhang X Yee D Insulin receptor substrates mediate distinct biological responses to insulin-like growth factor receptor activation in breast cancer cells Br J Cancer 2006 95 1220 1228 10.1038/sj.bjc.6603354 17043687 PMC2360584

[B75] Tang Q Chen Y Meyer C Geistlinger T Lupien M Wang Q Liu T Zhang Y Brown M Liu XS A comprehensive view of nuclear receptor cancer cistromes Cancer Res 2011 71 6940 6947 10.1158/0008-5472.CAN-11-2091 21940749 PMC3610570

[B76] Hager GL McNally JG Misteli T Transcription dynamics Mol Cell 2009 35 741 753 10.1016/j.molcel.2009.09.005 19782025 PMC6326382

[B77] Halford SE Marko JF How do site-specific DNA-binding proteins find their targets? Nucleic Acids Res 2004 32 3040 3052 10.1093/nar/gkh624 15178741 PMC434431

[B78] Carroll JS Liu XS Brodsky AS Li W Meyer CA Szary AJ Eeckhoute J Shao W Hestermann EV Geistlinger TR Fox EA Silver PA Brown M Chromosome-wide mapping of estrogen receptor binding reveals long-range regulation requiring the forkhead protein FoxA1 Cell 2005 122 33 43 10.1016/j.cell.2005.05.008 16009131

[B79] Lupien M Eeckhoute J Meyer CA Wang Q Zhang Y Li W Carroll JS Liu XS Brown M FoxA1 translates epigenetic signatures into enhancer-driven lineage-specific transcription Cell 2008 132 958 970 10.1016/j.cell.2008.01.018 18358809 PMC2323438

[B80] Hurtado A Holmes KA Ross-Innes CS Schmidt D Carroll JS FOXA1 is a key determinant of estrogen receptor function and endocrine response Nat Genet 2011 43 27 33 10.1038/ng.730 21151129 PMC3024537

[B81] Man J-H Li H-Y Zhang P-J Zhou T He K Pan X Liang B Li A-L Zhao J Gong W-L Jin B-F Xia Q Yu M Shen B-F Zhang X-M PIAS3 induction of PRB sumoylation represses PRB transactivation by destabilizing its retention in the nucleus Nucleic Acids Res 2006 34 5552 5566 10.1093/nar/gkl691 17020914 PMC1635300

[B82] Holmstrom SR Chupreta S So AY Iñiguez-Lluhí JA SUMO-mediated inhibition of glucocorticoid receptor synergistic activity depends on stable assembly at the promoter but not on Daxx Mol Endocrinol 2008 22 2061 2075 10.1210/me.2007-0581 18562626 PMC2631372

[B83] Heintzman ND Stuart RK Hon G Fu Y Ching CW Hawkins RD Barrera LO Van Calcar S Qu C Ching KA Wang W Weng Z Green RD Crawford GE Ren B Distinct and predictive chromatin signatures of transcriptional promoters and enhancers in the human genome Nat Genet 2007 39 311 318 10.1038/ng1966 17277777

[B84] ENCODE Project Consortium Identification and analysis of functional elements in 1% of the human genome by the ENCODE pilot project Nature 2007 447 799 816 10.1038/nature05874 17571346 PMC2212820

[B85] Meijsing SH Pufall MA So AY Bates DL Chen L Yamamoto KR DNA binding site sequence directs glucocorticoid receptor structure and activity Science 2009 324 407 410 10.1126/science.1164265 19372434 PMC2777810

[B86] Blind RD Garabedian MJ Differential recruitment of glucocorticoid receptor phospho-isoforms to glucocorticoid-induced genes J Steroid Biochem Mol Biol 2008 109 150 157 10.1016/j.jsbmb.2008.01.002 18304804 PMC2699583

[B87] Early Breast Cancer Trialists' Collaborative Group Effects of chemotherapy and hormonal therapy for early breast cancer on recurrence and 15-year survival: an overview of the randomised trials Lancet 2005 365 1687 1717 15894097 10.1016/S0140-6736(05)66544-0

[B88] Goss PE Ingle JN Ales-Martinez JE Cheung AM Chlebowski RT Wactawski-Wende J McTiernan A Robbins J Johnson KC Martin LW Winquist E Sarto GE Garber JE Fabian CJ Pujol P Maunsell E Farmer P Gelmon KA Tu D Richardson H Exemestane for breast-cancer prevention in postmenopausal women New Engl J Med 2011 364 2381 2391 10.1056/NEJMoa1103507 21639806

[B89] Kaufman B Mackey JR Clemens MR Bapsy PP Vaid A Wardley A Tjulandin S Jahn M Lehle M Feyereislova A Revil C Jones A Trastuzumab plus anastrozole versus anastrozole alone for the treatment of postmenopausal women with human epidermal growth factor receptor 2-positive, hormone receptor-positive metastatic breast cancer: results from the randomized phase III TAnDEM study J Clin Oncol 2009 27 5529 5537 10.1200/JCO.2008.20.6847 19786670

[B90] Johnston S Pippen J Jr Pivot X Lichinitser M Sadeghi S Dieras V Gomez HL Romieu G Manikhas A Kennedy MJ Press MF Maltzman J Florance A O'Rourke L Oliva C Stein S Pegram M Lapatinib combined with letrozole versus letrozole and placebo as first-line therapy for postmenopausal hormone receptor-positive metastatic breast cancer J Clin Oncol 2009 27 5538 5546 10.1200/JCO.2009.23.3734 19786658

[B91] Daniel AR Hagan CR Lange CA SUMO-mediated inhibition of glucocorticoid receptor synergistic activity depends on stable assembly at the promoter but not on Daxx Expert Rev Endocrinol Metab 2011 6 359 369 10.1586/eem.11.25 21857868 PMC3156468

[B92] Suzuki T Miki Y Nakamura Y Moriya T Ito K Ohuchi N Sasano H Sex steroid-producing enzymes in human breast cancer Endocr Relat Cancer 2005 12 701 720 10.1677/erc.1.00834 16322318

[B93] Locke JA Guns ES Lubik AA Adomat HH Hendy SC Wood CA Ettinger SL Gleave ME Nelson CC Androgen levels increase by intratumoral de novo steroidogenesis during progression of castration-resistant prostate cancer Cancer Res 2008 68 6407 6415 10.1158/0008-5472.CAN-07-5997 18676866

[B94] Su B Wong C Hong Y Chen S Growth factor signaling enhances aromatase activity of breast cancer cells via post-transcriptional mechanisms J Steroid Biochem Mol Biol 2011 123 101 108 10.1016/j.jsbmb.2010.11.012 21112394 PMC3030665

[B95] Amit I Citri A Shay T Lu Y Katz M Zhang F Tarcic G Siwak D Lahad J Jacob-Hirsch J Amariglio N Vaisman N Segal E Rechavi G Alon U Mills GB Domany E Yarden Y A module of negative feedback regulators defines growth factor signaling Nat Genet 2007 39 503 512 10.1038/ng1987 17322878

[B96] Chlebowski RT Anderson GL Gass M Lane DS Aragaki AK Kuller LH Manson JE Stefanick ML Ockene J Sarto GE Johnson KC Wactawski-Wende J Ravdin PM Schenken R Hendrix SL Rajkovic A Rohan TE Yasmeen S Prentice RL Estrogen plus progestin and breast cancer incidence and mortality in postmenopausal women JAMA 2010 304 1684 1692 10.1001/jama.2010.1500 20959578 PMC5142300

[B97] Musgrove EA Sutherland RL Biological determinants of endocrine resistance in breast cancer Nat Rev Cancer 2009 9 631 643 10.1038/nrc2713 19701242

[B98] Salatino M Schillaci R Proietti CJ Carnevale R Frahm I Molinolo AA Iribarren A Charreau EH Elizalde PV Inhibition of in vivo breast cancer growth by antisense oligodeoxynucleotides to type I insulin-like growth factor receptor mRNA involves inactivation of ErbBs, PI-3K/Akt and p42/p44 MAPK signaling pathways but not modulation of progesterone receptor activity Oncogene 2004 23 5161 5174 10.1038/sj.onc.1207659 15122317

[B99] Labriola L Salatino M Proietti CJ Pecci A Coso OA Kornblihtt AR Charreau EH Elizalde PV Heregulin induces transcriptional activation of the progesterone receptor by a mechanism that requires functional ErbB-2 and mitogen-activated protein kinase activation in breast cancer cells Mol Cell Biol 2003 23 1095 1111 10.1128/MCB.23.3.1095-1111.2003 12529413 PMC140689

[B100] Parker JS Mullins M Cheang MCU Leung S Voduc D Vickery T Davies S Fauron C He X Hu Z Quackenbush JF Stijleman IJ Palazzo J Marron JS Nobel AB Mardis E Nielsen TO Ellis MJ Perou CM Bernard PS Supervised risk predictor of breast cancer based on intrinsic subtypes J Clin Oncol 2009 27 1160 1167 10.1200/JCO.2008.18.1370 19204204 PMC2667820

[B101] Paik S Shak S Tang G Kim C Baker J Cronin M Baehner FL Walker MG Watson D Park T Hiller W Fisher ER Wickerham DL Bryant J Wolmark N A multigene assay to predict recurrence of tamoxifen-treated, node-negative breast cancer New Engl J Med 2004 351 2817 2826 10.1056/NEJMoa041588 15591335

[B102] van't Veer LJ Dai H van de Vijver MJ He YD Hart AA Mao M Peterse HL van der Kooy K Marton MJ Witteveen AT Schreiber GJ Kerkhoven RM Roberts C Linsley PS Bernards R Friend SH Gene expression profiling predicts clinical outcome of breast cancer Nature 2002 415 530 536 10.1038/415530a 11823860

