# Supplementary figures and images for: Phosphorylated and sumoylation-deficient progesterone receptors drive proliferative gene signatures during breast cancer progression
Source: Breast Cancer Res. 2012 Jun 14;14(3):R95. doi: 10.1186/bcr3211 (PMC3446358; doi:10.1186/bcr3211)

A

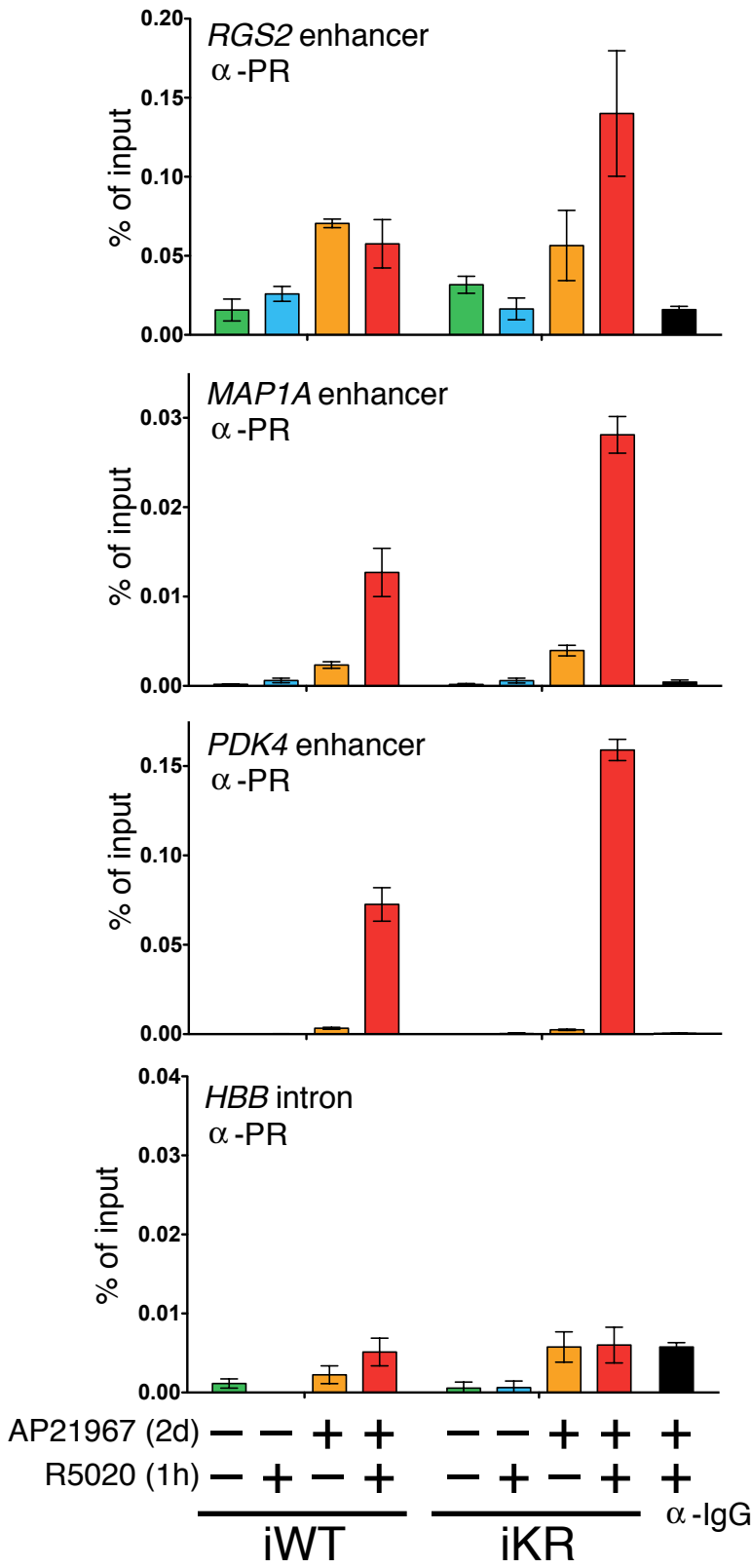

B

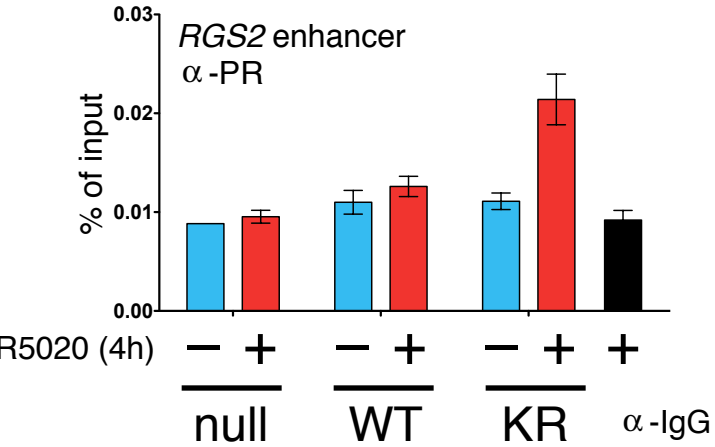

Supplement: Additional file 4 — Relative recruitment of WT and SUMO-deficient PR molecules to selected PR target gene enhancers. (A) Recruitment of PR molecules to consensus PRE sequences in upstream promoter/enhancer regions of RGS2, MAP1A, and PDK4 (following one hour R5020) was measured by standard ChIP assay in inducible models of T47D cells expressing WT (iWT) and KR (iKR) receptors. Recruitment of PR to an intronic region of the HBB gene was included as a negative control. (B) ChIP assays were performed as in part A, to demonstrate differential PR recruitment to a RGS2 enhancer in T47D cells stably expressing either WT or SUMO-deficient (KR) PR. Data are represented as mean of n = 3 +/- SD. [file bcr3211-S4.PDF]

A

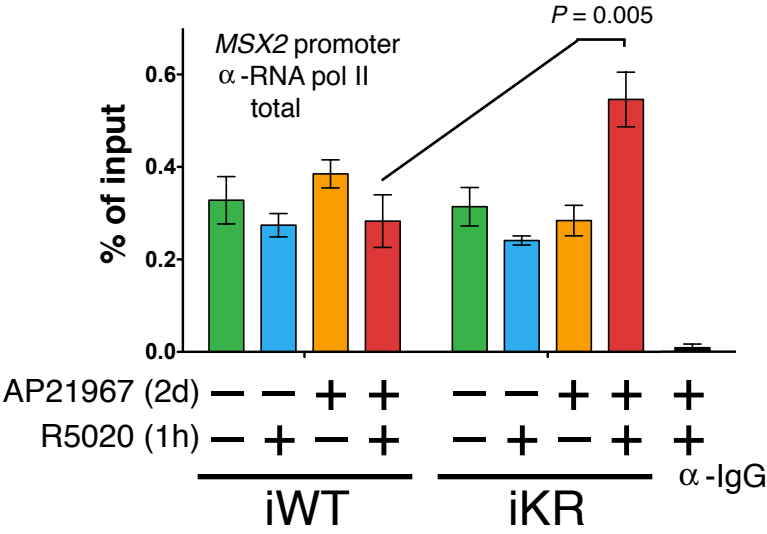

B

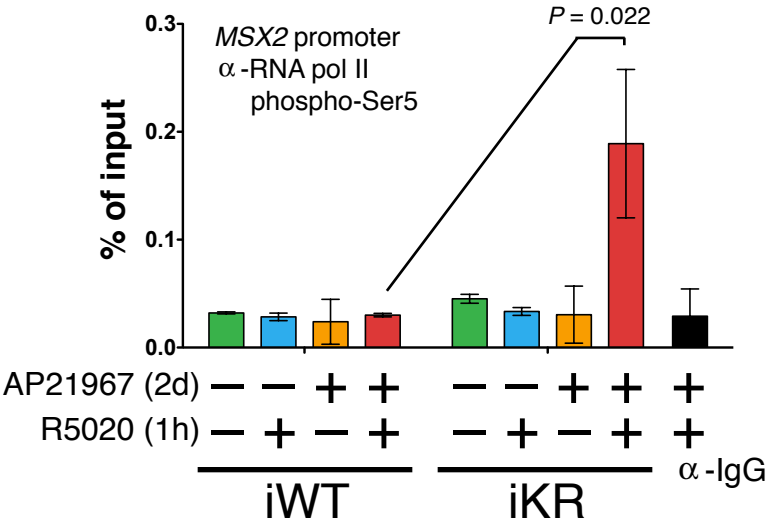

Supplement: Additional file 5 — Recruitment of phospho-Ser5 and total-RNA polymerase II to the MSX2 proximal promoter region. (A) Recruitment of total RNA polymerase II to the MSX2 proximal promoter region (following one hour R5020) was measured by standard ChIP assay in inducible models of T47D cells expressing WT (iWT) and KR (iKR) receptors. (B) ChIP assay was performed as in part A, using an antibody targeting functionally active RNA polymerase II, as measured by detection of CTD Ser5 phosphorylation. Data are represented as mean of n = 3 +/- SD. [file bcr3211-S5.PDF]

Ingenuity Pathway Analysis

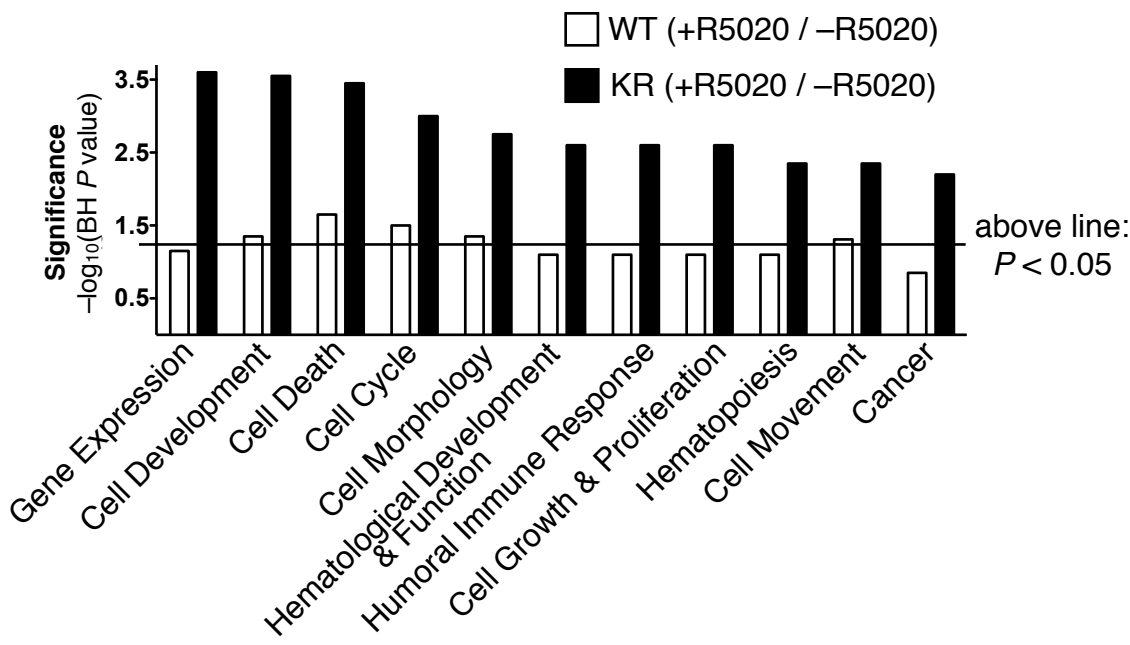

Supplement: Additional file 6 — SUMO-deficient PR upregulates genes involved in cell proliferation determined by Ingenuity Pathway Analysis. Significant expression (y-axis) of multiple cellular functions (x-axis) containing genes upregulated by progestin (log2 fold change > 1.0, BH adjusted P < 0.01; common fold change > 2.0) in cells expressing either WT or KR PR. Biological pathways that contain a significant number of upregulated genes display bars above the horizontal line, representing BH adjusted P < 0.05. [file bcr3211-S6.PDF]
