# Supplementary material for: Phosphorylated and sumoylation-deficient progesterone receptors drive proliferative gene signatures during breast cancer progression
Source: Breast Cancer Res. 2012 Jun 14;14(3):R95. doi: 10.1186/bcr3211 (PMC3446358; doi:10.1186/bcr3211)
Supplement: Additional file 2 — Creation and validation of isogenic models of inducible PR expression in T47D cells. (A) Clonal inducible cell lines were developed as described in the Materials and methods section and PR protein expression was determined by western blotting after treatment with inducer molecule AP21967 for two days and R5020 for one hour. Progestin-dependent PR phosphorylation was measured using a PR phospho-Ser294 specific antibody. Beta-actin western blotting was performed as a loading control. Short-term treatment with R5020 demonstrated progestin-dependent PR global phosphorylation (as indicated by a slight gel upshift in total PR) and equal levels of ligand-dependent Ser294 phosphorylation. (B) Gene set enrichment analysis (GSEA) comparison of whole genome expression profiling data sets derived from two independent model systems and platforms: (i) T47D cells stably expressing WT and mutant KR PRs (-/+R5020) using the Illumina HT-12v4 platform and (ii) T47D cells expressing inducible WT or mutant KR PR (-/+AP21967, -/+R5020) using the Affymetrix U133A 2.0 platform. Genes most upregulated in the Illumina dataset by WT +R5020 (or KR +R5020) appear on the far left (darkest red) and genes most downregulated by WT +R5020 (or KR +R5020) appear on the far right side (darkest blue). Using the GSEA application, Affymetrix genes (black vertical bars) were positioned along the Illumina dataset (from upregulated to downregulated genes) and the statistical enrichment score was determined. All the treatment groups between Affymetrix and Illumina were statistically significant (P < 0.001). (C) Gene expression levels were validated for two PR target genes (MSX2 and MAP1A) in T47D cell lines expressing iWT and iKR PR. Cells were treated with AP21967 to induce PR expression and co-treated with RU486 and/or R5020 before RT-qPCR gene expression analysis. Data are represented as mean of n = 3 +/- SD. [file bcr3211-S2.PDF]

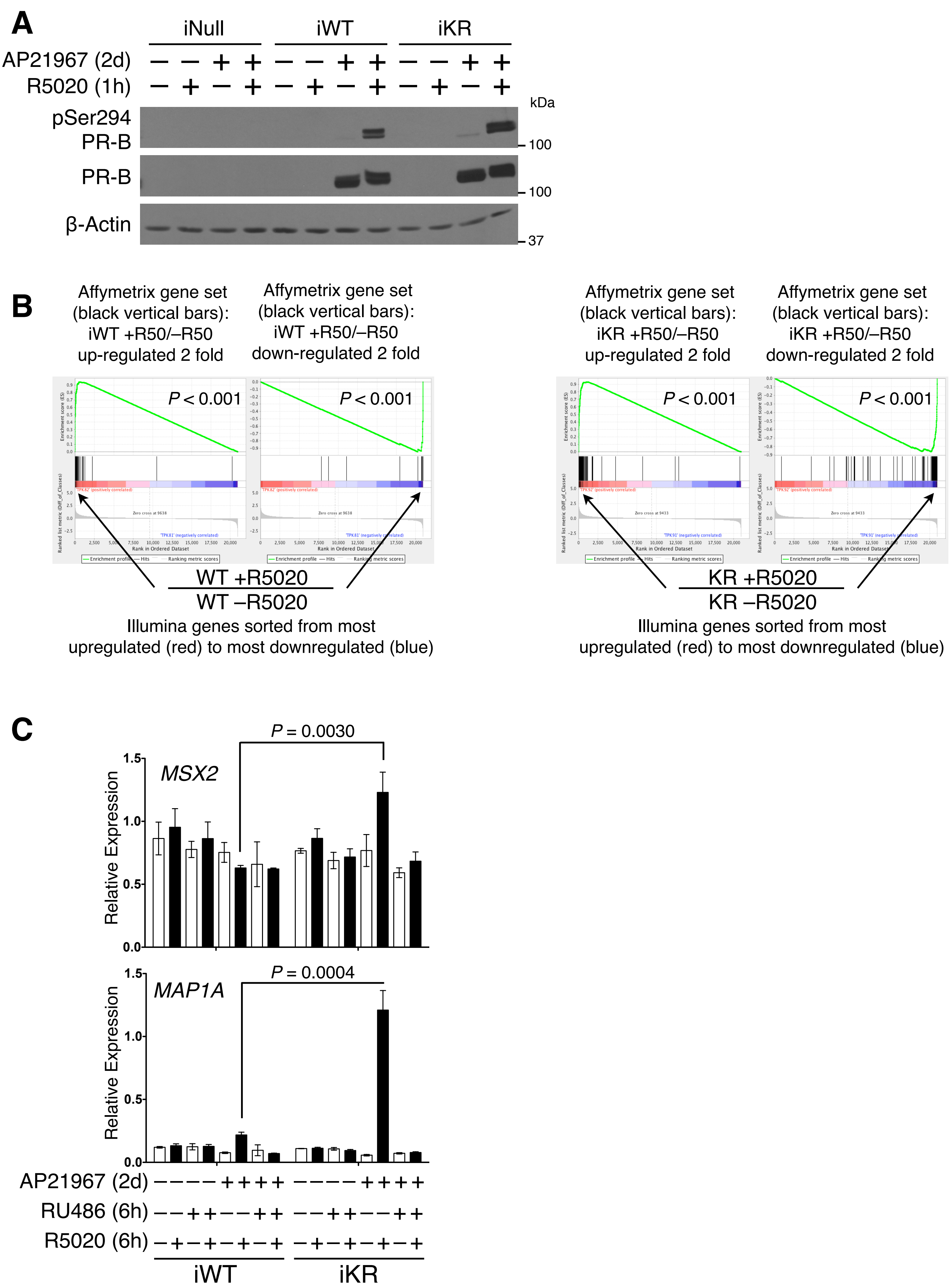
A
iNull
iWT
iKR
AP21967 (2d) − − + + − − + + − − + +
R5020 (1h) − + − + − + − + − + − +
kDa
pSer294 PR-B
100
PR-B
100
β-Actin
37
B
Affymetrix gene set (black vertical bars): iWT +R50/–R50 up-regulated 2 fold
Affymetrix gene set (black vertical bars): iWT +R50/–R50 down-regulated 2 fold
Affymetrix gene set (black vertical bars): iKR +R50/–R50 up-regulated 2 fold
Affymetrix gene set (black vertical bars): iKR +R50/–R50 down-regulated 2 fold
P < 0.001
P < 0.001
P < 0.001
P < 0.001
WT +R5020 / WT –R5020
KR +R5020 / KR –R5020
Illumina genes sorted from most upregulated (red) to most downregulated (blue)
Illumina genes sorted from most upregulated (red) to most downregulated (blue)
C
P = 0.0030
MSX2
Relative Expression
1.5
1.0
0.5
0.0
P = 0.0004
MAP1A
Relative Expression
1.5
1.0
0.5
0.0
AP21967 (2d) − − − − + + + + − − − − + + + +
RU486 (6h) − − + + − − + + − − + + − − + +
R5020 (6h) − + − + − + − + − + − + − + − +
iWT
iKR
